# Combined Notch and PDGF Signaling Enhances Migration and Expression of Stem Cell Markers while Inducing Perivascular Cell Features in Muscle Satellite Cells

**DOI:** 10.1016/j.stemcr.2019.01.007

**Published:** 2019-02-07

**Authors:** Mattia Francesco Maria Gerli, Louise Anne Moyle, Sara Benedetti, Giulia Ferrari, Ekin Ucuncu, Martina Ragazzi, Chrystalla Constantinou, Irene Louca, Hiroshi Sakai, Pierpaolo Ala, Paolo De Coppi, Shahragim Tajbakhsh, Giulio Cossu, Francesco Saverio Tedesco

**Affiliations:** 1 Department of Cell and Developmental Biology, University College London, WC1E 6DE London, UK; 2 Stem Cell and Regenerative Medicine Section, Great Ormond Street Institute of Child Health, University College London, WC1N 1EH London, UK; 3 Molecular and Cellular Immunology Section, Great Ormond Street Institute of Child Health, University College London, WC1N 1EH London, UK; 4 NIHR Great Ormond Street Hospital Biomedical Research Centre, WC1N 1EH London, UK; 5 Department of Developmental & Stem Cell Biology, Institut Pasteur, 75015 Paris, France; 6 CNRS UMR 3738, Institut Pasteur, 75015 Paris, France; 7 The Dubowitz Neuromuscular Centre, Great Ormond Street Institute of Child Health, University College London, WC1N 1EH London, UK; 8 Division of Cell Matrix Biology and Regenerative Medicine, University of Manchester, M13 9PL Manchester, UK

**Keywords:** muscle stem cells, satellite cells, stem cell fate, reprogramming, perivascular cells, muscle regeneration, muscular dystrophy, cell therapy, NOTCH, PDGF

## Abstract

Satellite cells are responsible for skeletal muscle regeneration. Upon activation, they proliferate as transient amplifying myoblasts, most of which fuse into regenerating myofibers. Despite their remarkable differentiation potential, these cells have limited migration capacity, which curtails clinical use for widespread forms of muscular dystrophy. Conversely, skeletal muscle perivascular cells have less myogenic potential but better migration capacity than satellite cells. Here we show that modulation of Notch and PDGF pathways, involved in developmental specification of pericytes, induces perivascular cell features in adult mouse and human satellite cell-derived myoblasts. DLL4 and PDGF-BB-treated cells express markers of perivascular cells and associate with endothelial networks while also upregulating markers of satellite cell self-renewal. Moreover, treated cells acquire trans-endothelial migration ability while remaining capable of engrafting skeletal muscle upon intramuscular transplantation. These results extend our understanding of muscle stem cell fate plasticity and provide a druggable pathway with clinical relevance for muscle cell therapy.

## Highlights

• DLL4 and PDGF-BB change satellite cell morphology, proliferation, and differentiation
• DLL4 and PDGF-BB induce both perivascular and satellite cell gene expression
• Treated satellite cells acquire perivascular cell properties and improved migration
• Human satellite cell-derived myoblasts respond to DLL4 and PDGF-BB treatment

## Graphical Abstract

**Figure:**
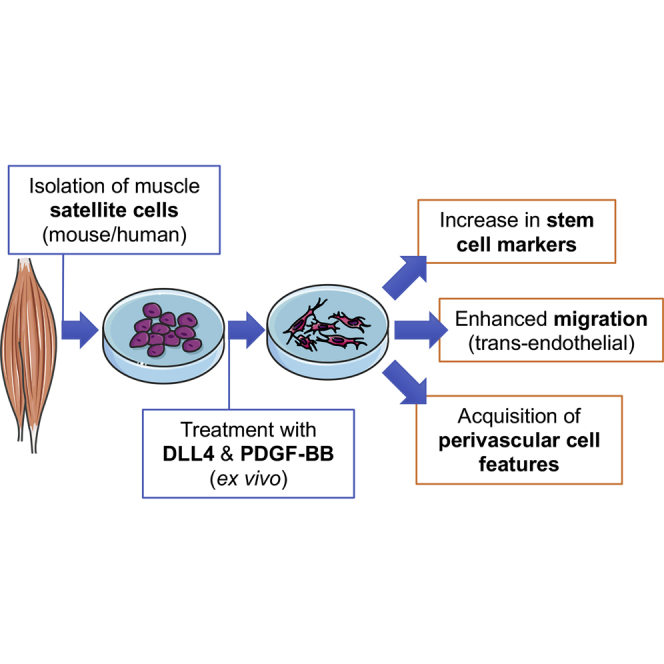

## Teaser

Gerli and Moyle and colleagues show that treatment with molecules involved in developmental specification of pericytes (DLL4 and PDGF-BB) alters satellite cell fate and provides them with features potentially relevant for novel cell therapy protocols.

## Introduction

Skeletal muscle homeostasis and regeneration rely on resident stem cells named satellite cells (SCs), which reside underneath the basal lamina of the myofibers and express the transcription factor *Pax7*. Upon injury, activated SCs generate transient amplifying precursors called myoblasts, which fuse to form multinucleated myofibers (Sambasivan and Tajbakhsh, 2015). The regenerative capacity of SCs has led to the development of cellular therapies to replace lost or damaged muscle (Tedesco et al., 2010). Despite some promising pre-clinical results and a good safety profile, clinical trials based upon intramuscular myoblast transplantation in patients with Duchenne muscular dystrophy (DMD) have reported limited efficacy (Briggs and Morgan, 2013). This outcome has been ascribed to the poor survival of myoblasts, their limited ability to migrate, and the host immune reaction (Partridge, 2002), although this is still a matter of active debate (Skuk and Tremblay, 2014). More recently, local delivery of myoblasts to affected muscles in oculopharyngeal muscular dystrophy patients has shown encouraging results (Perie et al., 2014). Nevertheless, myoblasts are considered unsuitable for systemic delivery, preventing their use for the treatment of patients affected by severe myopathies with widespread muscle involvement such as DMD.

Perivascular cells (pericytes in particular) support skeletal muscle perfusion, development, and regeneration (Birbrair and Delbono, 2015, Cappellari and Cossu, 2013, Murray et al., 2017). Despite evidence in transgenic mice showing that SCs (Relaix and Zammit, 2012) and not pericytes (Guimaraes-Camboa et al., 2017) are required for muscle regeneration, there are reports indicating that pericyte-derived cells can also contribute to skeletal myogenesis, including SC generation and maintenance (Dellavalle et al., 2011, Dellavalle et al., 2007, Kostallari et al., 2015, Sacchetti et al., 2016, Tedesco et al., 2011). Discrepancies among reports may be due to the use of different markers to identify interstitial/perivascular cells (Tedesco et al., 2017). Importantly, intra-arterial delivery of progenitors derived from skeletal muscle perivascular cells (mainly mesoangioblasts, deriving from *in vitro* expansion of a subset of muscle pericytes) resulted in the colonization of skeletal muscle tissue downstream of the injection site and subsequent amelioration of different animal models of muscular dystrophy (Benedetti et al., 2013). Moreover, a recent first-in-human phase I/IIa clinical trial based on intra-arterial delivery of human leukocyte antigen-matched mesoangioblasts in DMD children has established the safety and feasibility of this procedure (Cossu et al., 2015). While they may be an important source for transplantation, the skeletal myogenic and self-renewing potential of perivascular cells is suboptimal compared with SCs, and their preliminary clinical investigation indicates that further optimization will be needed for muscle cell therapy (Cossu et al., 2015). Therefore, a muscle stem cell harboring SC myogenic and self-renewing capacity combined with the migration ability of perivascular cells could be ideal for muscle cell therapies.

Several groups have shown that the Notch signaling pathway, a key regulator of myogenesis and pericyte function, can alter the behavior of myogenic precursors (Mourikis and Tajbakhsh, 2014, Sainson and Harris, 2008). The Notch ligand delta ligand 1 (DLL1) promotes SC quiescence (Baghdadi et al., 2018) and increases engraftment of canine muscle cells (Parker et al., 2012), whereas DLL4 regulates mouse SC self-renewal (Low et al., 2018, Verma et al., 2018); however, DLL1 and DLL4 alone did not significantly improve engraftment of mouse and human SCs (Sakai et al., 2017). Conversely, Notch depletion leads to SC exhaustion, impairment of muscle regeneration, and reduced engraftment of mesoangioblasts (Bjornson et al., 2012, Mourikis et al., 2012, Quattrocelli et al., 2014, Schuster-Gossler et al., 2007, Vasyutina et al., 2007).

Platelet-derived growth factor (PDGF) signaling also has important roles in regulating smooth and skeletal muscle cell fate. The PDGF signaling pathway comprises the two receptors α (PDGFR-A) and β (PDGFR-B), which bind to ligands PDGF-A/-B/-C/-D as homo- or hetero-dimers (Lu and Li, 2017). PDGF-B is expressed in both SC and pericytes (Pinol-Jurado et al., 2017), affecting their proliferation, migration, recruitment, and fate (Lindahl et al., 1997, Pallafacchina et al., 2010, Sugg et al., 2017, Yablonka-Reuveni et al., 1990). In addition, PDGF-BB is upregulated in dystrophic myofibers and attracts myoblasts (Pinol-Jurado et al., 2017); with a similar mechanism, endothelial cells recruit mural cells via PDGF-BB (Betsholtz, 2004). Importantly, Notch induces PDGFR-B, and this combined signaling directs vascular smooth muscle cell fate choice (Jin et al., 2008).

Previously we reported that mouse embryonic myoblasts undergo a fate switch toward the perivascular lineage following stimulation with DLL4 and PDGF-BB (Cappellari et al., 2013). Although this prior study suggests bidirectional fate plasticity between SCs and pericytes, there is currently no evidence indicating that a similar phenomenon is conserved in adult myogenic progenitors. Here, we provide evidence that adult skeletal muscle SCs gain pericyte properties in response to DLL4 and PDGF-BB treatment, while also re-acquiring a stemness signature.

## Results

### DLL4 and PDGF-BB Treatment Induces Reversible Changes in Morphology, Proliferation, and Differentiation of Adult Murine Satellite Cell-Derived Myoblasts

**Figure 1:**
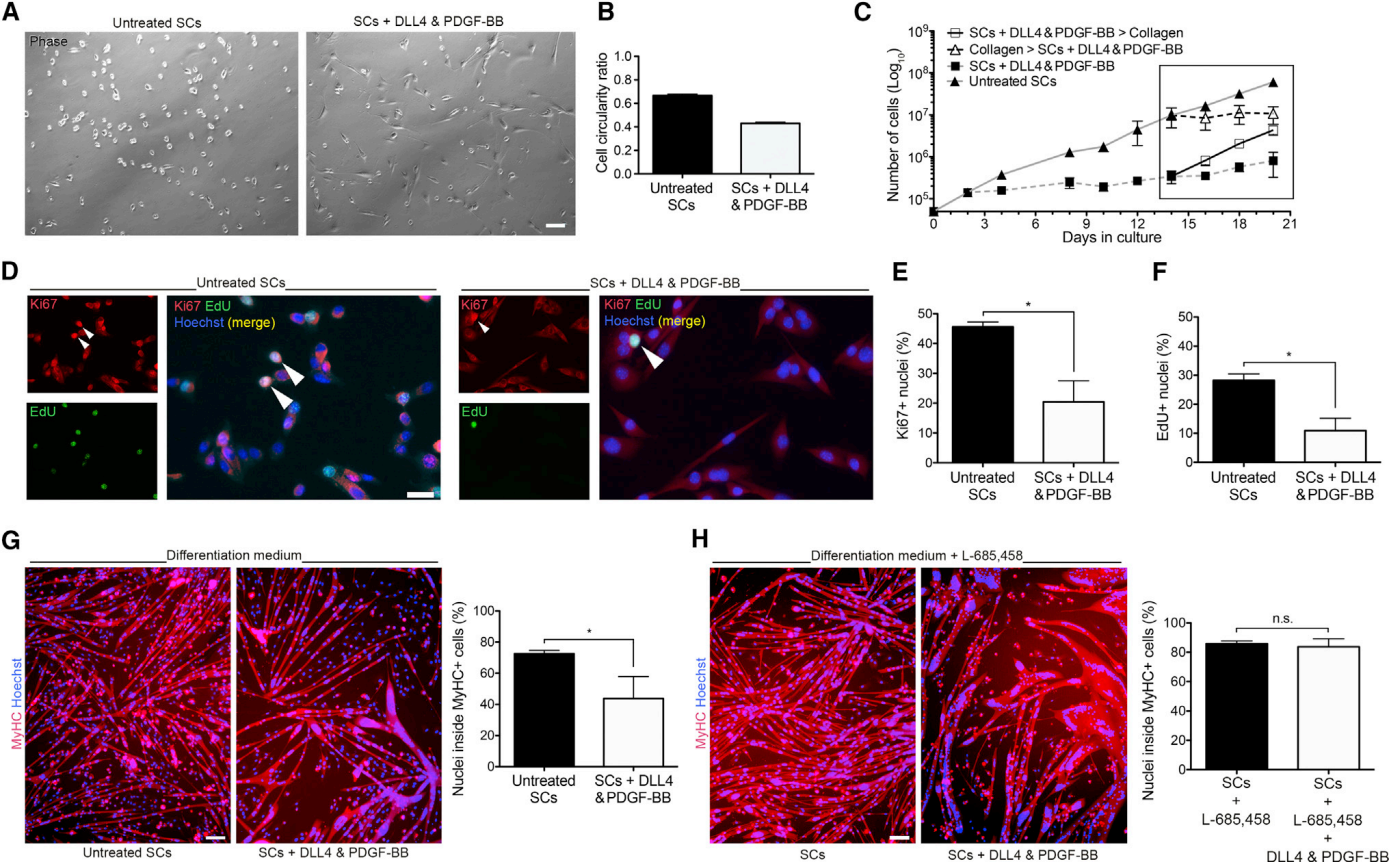
Morphology, Proliferation, and Differentiation of DLL4 and PDGF-BB-Treated SCs (A) Phase contrast images of untreated and DLL4 and PDGF-BB-treated SCs isolated from CD1 mice. (B) Graph quantifies circularity ratio, where 1 = circle and 0 = line (n = 3). (C) Proliferation curves of untreated and treated SCs over time (n = 3). Box highlights treatment switch. (D–F) Immunofluorescence analysis of SCs isolated from *TN-AP*^*cre*^*:TdTomato* mice expanded for 2 weeks prior to treatment, or maintained in untreated conditions. Cells pulsed for 2 h with EdU and co-immunostained with Ki67 (arrowheads: nuclear signal) (N = 3) (D). Quantified in (E and F). (G) Immunofluorescence images of untreated and treated SCs differentiated into myotubes in low mitogen medium for 4 days and immunostained for myosin heavy chain (MyHC) and Hoechst (N = 3 mice and 4 experiments). (H) Untreated and treated SCs differentiated in low mitogen medium supplemented with 660 ng/mL of the γ-secretase inhibitor L-685,458 to inhibit Notch signaling (N = 3). Data: means ± SEM. Statistical significance based on paired (E and F) or unpaired (G and H) Student's t test; ^∗^p < 0.05; n.s., not significant. Scale bars, 25 μm (D) and 100 μm (A, G, and H).

To determine whether adult SCs respond to the activation of Notch and PDGF pathways, primary SC-derived myoblast cultures (hereafter referred to as SCs) were established from wild-type mice (Figure S1A) and cultured on collagen-coated dishes (to aid attachment) or seeded on DLL4-coated dishes supplemented daily with PDGF-BB. After 1 week of treatment, the morphology of the treated SCs was compared with untreated control SCs, revealing a change from a round to a more elongated morphology (Figures 1A and 1B).

Notch activity controls SC activation, proliferation, and quiescence (Bjornson et al., 2012, Brohl et al., 2012, Mourikis et al., 2012). Consistent with these reports, we observed reduced proliferation of SCs exposed to the treatment (Figure 1C). This effect was reverted by discontinuation of the treatment. Interestingly, reduced cell proliferation was also observed when the treatment was commenced on cells that were previously expanded for 2 weeks in control conditions, indicating that the responsiveness to treatment was not impaired with extensive culture (Figures 1D–1F). Specifically, proliferation was analyzed by functional 5-ethynyl-2′-deoxyuridine (EdU) incorporation assay and nuclear Ki67 immunofluorescence staining. Both assays showed a decrease in proliferation from control to treated SCs: 28.19% vs 10.94% EdU incorporation (p = 0.021, N = 3) and 45.60% vs 20.44% Ki67 positivity (p = 0.047, N = 3).

Notch activation inhibits myogenesis *in vitro* both in embryonic myoblasts and adult SCs (Conboy and Rando, 2002, Kopan et al., 1994, Mourikis and Tajbakhsh, 2014). To investigate if this mechanism is triggered upon treatment with DLL4 and PDGF-BB, cells were differentiated into skeletal myotubes and immunoassayed for myosin heavy chain (MyHC) (Figures 1G and 1H). A significant reduction in myogenic differentiation upon treatment was observed, from 71.5% to 37.9% (p = 0.031, N = 3 mice in 4 experiments), reflecting the role of Notch signaling in inhibiting myogenesis. This differentiation impairment was reverted by blocking the Notch cascade with the γ-secretase inhibitor L685,458 (N = 3), confirming the Notch dependency of this phenomenon (Figures 1G and 1H).

### DLL4 and PDGF-BB Treatment Induces Transcription of Perivascular and SC Genes

To understand the mechanism behind the observed proliferation and differentiation changes, a series of real-time qPCR analyses was performed to investigate the variation in the gene expression profile of SCs upon DLL4 and PDGF-BB treatment. Preliminary experiments performed with SCs isolated with the pre-plating methodology from CD1 mice (Figure S1A) suggested that a number of transcripts associated with Notch and PDGF pathways, as well as myogenic and smooth muscle programs, responded to treatment (Figure S1B).

**Figure 2:**
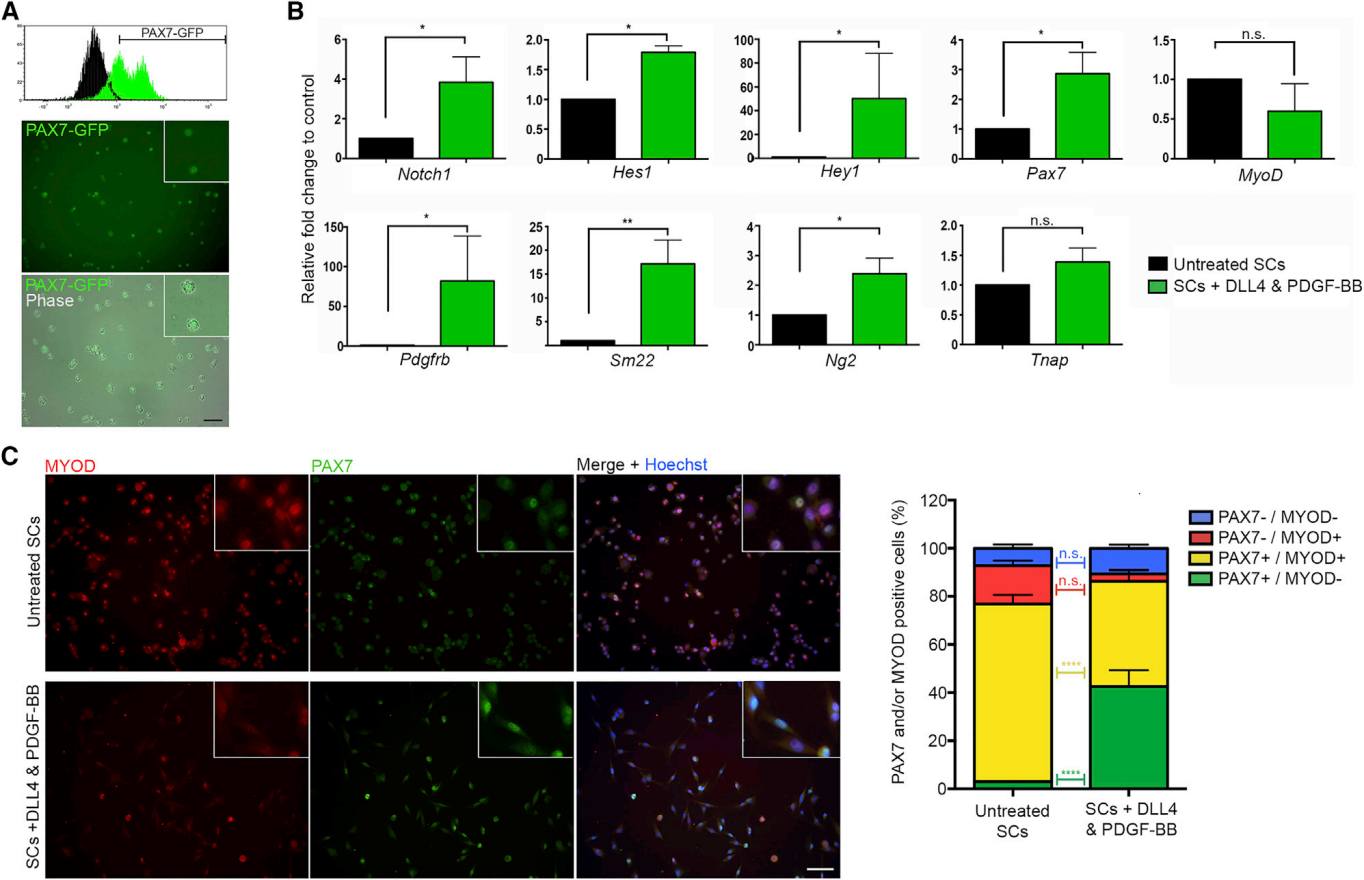
Gene Expression Analyses of Treated SCs via Real-Time qPCR and Immunofluorescence (A) Histogram view of the FACS purification of PAX7-GFP+ SCs and resulting pure population in phase contrast and GFP images. (B) Real-time qPCR analysis of untreated (black bars) and DLL4 and PDGF-BB-treated (green bars) PAX7-GFP+ SCs. Graphs show fold change to control conditions, statistical significance based on ΔCt (N = 4). (C) Left panel: PAX7-GFP+ SCs cultured in control (untreated) or DLL4 and PDGF-BB conditions and immunostained with MYOD (red) and PAX7 (green) antibodies. Right graph quantifying the relative percentages of PAX7/MYOD +/− cells in each condition (N = 3). Data: means ± SEM. Statistical significance based on paired Student's t tests (B) or two-way ANOVA with Bonferroni's multiple comparison (C). ^∗^p ≤ 0.05, ^∗∗^p ≤ 0.01, ^∗∗∗∗^p ≤ 0.0001, n.s., not significant. Scale bars, 50 μm (A and C). See also Figure S1.

To ensure that the observed gene expression profile was directly elicited by DLL4 and PDGF-BB on bona fide SCs and not on a possible minority of contaminating cells, we used SCs isolated from *Tg:Pax7-nGFP* mice (Sambasivan et al., 2009) (Figure 2A). Analysis of the expression of *Notch1*, the main receptor for DLL4, and of *Pdgfrb*, showed significant transcriptional upregulation, confirming that treatment with DLL4 and PDGF-BB activated the expected signaling pathways (p = 0.038 and p = 0.017, respectively, Figure 2B). This was also confirmed by increased expression of Notch targets *Hes1* (p = 0.028) and *Hey1* (p = 0.022). Notch induces *Pdgfrb* expression in vascular smooth muscle cells (Jin et al., 2008), which may explain the remarkable induction of *Pdgfrb*. Interestingly, the treatment induced genes usually more expressed in smooth muscle cells and pericytes, including *Ng2* (p = 0.024) and *Sm22* (p = 0.003), while also increasing expression of the SC marker *Pax7* (p = 0.032). Finally, quantification of alkaline phosphatase (AP) activity, which is associated with myogenic pericytes (Dellavalle et al., 2011), suggested upregulation in treated cells (Figure S1C).

We then measured PAX7 and MYOD proteins in SCs treated with DLL4 and PDGF-BB (Figure 2C, N = 3). PAX7 marks quiescent, self-renewing, and primed SCs, whereas MYOD is associated with committed progenitors (i.e., myoblasts) (Zammit et al., 2004). Similar to what was observed at the mRNA level, the percentage of PAX7+/MYOD– cells increased from 2.97% in untreated cells to 42.47% in treated cells (p < 0.0001). Conversely, the percentage of PAX7+/MYOD+ SCs decreased with DLL4 and PDGF-BB treatment, from 73.90% to 43.79% (p < 0.0001), suggesting that treated cells retained a more “stem-like” state.

For this treatment to be applicable to human cells, response needs to be maintained following expansion in culture. To test this, we assessed whether SCs were responsive to treatment when expanded for 2 weeks prior to treatment, revealing an increase in the proportion of PAX7+/MYOD– SCs (p = 0.001, N = 4; Figure S1D).

Finally, we observed that the morphological changes elicited in treated SCs correlated to an ability of the cells to adhere directly to plastic, rather than requiring collagen coating. To confirm that the effect of DLL4 and PDGF-BB treatment was independent of collagen coating, SCs from *Tg:Pax7-nGFP* mice were expanded for 1 week in control and treatment conditions on collagen-coated or uncoated culture dishes: proliferation kinetics and mRNA expression analyses revealed that the effect was independent of coating (Figures S1E and S1F; N = 3). Taken together, these results show that DLL4 and PDGF-BB treatment induces a perivascular-like gene expression profile in SCs while maintaining or inducing a signature associated with their self-renewal.

### DLL4 and PDGF-BB-Treated SCs Gain Properties of Perivascular Cells

**Figure 3:**
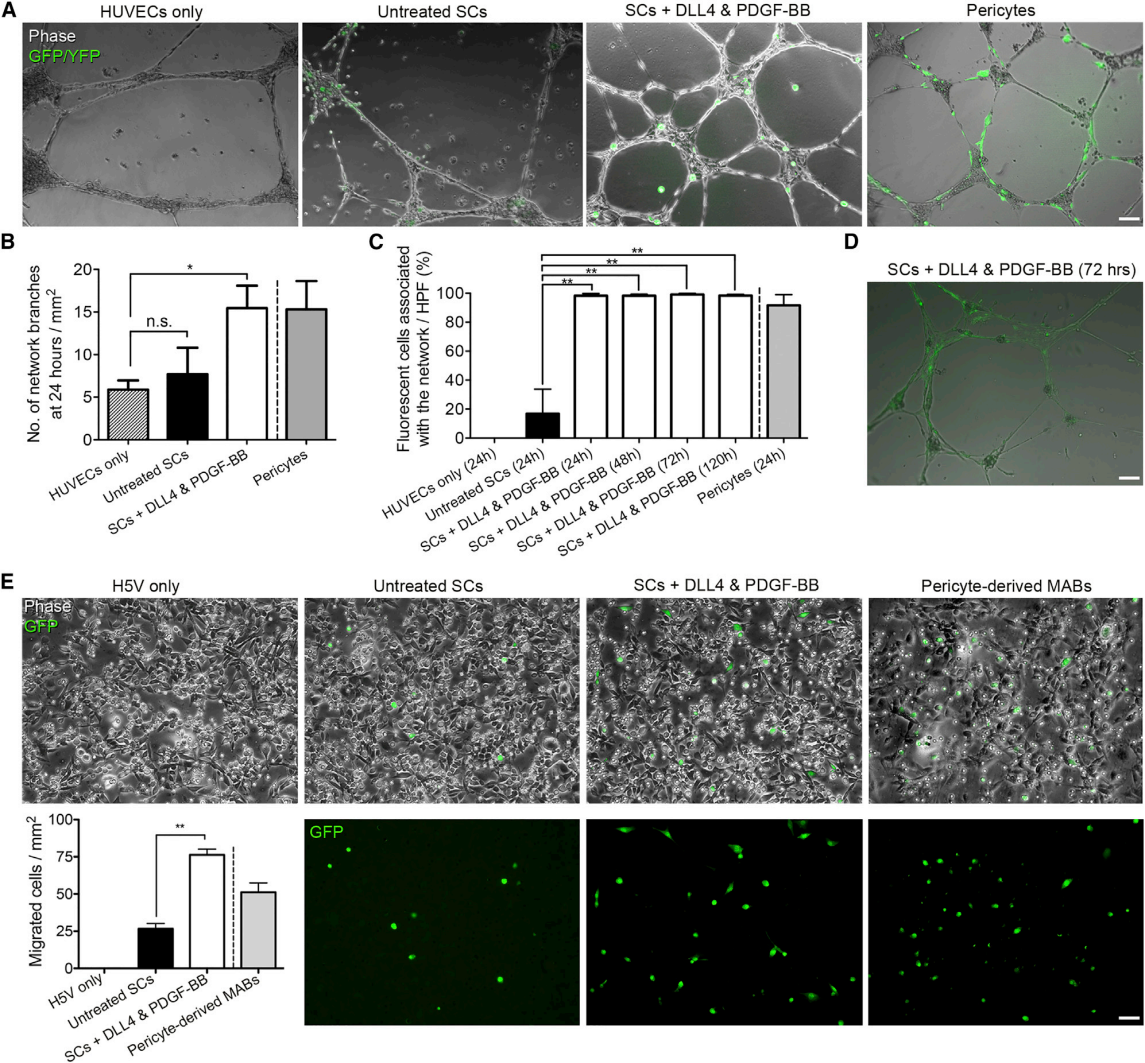
*In Vitro* Assessment of Endothelial Network Association and Migration Properties of DLL4 and PDGF-BB-Treated SCs (A) Representative images from endothelial network formation assays at 24 h with GFP+ untreated or DLL4 and PDGF-BB-treated SCs and control yellow fluorescent protein (YFP)+ primary muscle pericytes (technical control, from *Tg:TN-AP-CreERT2*:*R2*6R*-EYFP* mice) stimulated with VEGF in the presence of HUVECs. (B) Graph depicts quantification of network branches per mm^2^ at 24 h in the same experimental groups shown in (A) (N = 4). (C and D) Graph quantifies GFP+ SCs and YFP+ pericytes associated with the network per high-power field (HPF = 1.5 mm^2^) over time (N = 4); representative image of treated cells at 72 h shown in (D). (E) Images of the lower side of transwell membranes from GFP+ untreated or treated SCs and control pericyte-derived mesoangioblasts (MABs) seeded on a monolayer of H5V murine endothelial cells. Graph quantifies the average number of cells/mm^2^ that migrated through the endothelial layer after 6 h (N = 3). Means ± SEM; statistical significance based on one-way ANOVA with Dunnett's multiple comparison test (B and C) or paired t test (E); ^∗^p < 0.05, ^∗∗^p ≤ 0.01. Scale bars, 100 μm.

We next aimed to test whether the expression of perivascular markers on SC treatment with DLL4 and PDGF-BB was also accompanied by functional pericyte properties. Pericytes contribute to the stabilization of endothelial networks in hydrogels on vascular endothelial growth factor (VEGF) stimulation (Cappellari et al., 2013). To determine whether DLL4 and PDGF-BB treatment conferred this functional property to SCs, treated cells were co-cultured with human umbilical vein endothelial cells (HUVECs) in Matrigel and stimulated with VEGF. To allow cell tracing in co-culture, SCs were isolated from *Tg:CAG-EGFP* mice, which ubiquitously express the green fluorescent protein (GFP) (Okabe et al., 1997). After 24 h in culture, endothelial networks showed limited branching and partial disaggregation when cultured alone or with untreated SCs; however, networks showed increased branching and stability when co-cultured with SCs exposed to DLL4 and PDGF-BB, similarly to control skeletal muscle pericytes (Figures 3A–3D).

A characteristic of pericyte-derived mesoangioblasts is their ability to migrate outside blood vessels upon intravascular delivery in models of muscular dystrophy (e.g., Giannotta et al., 2014, Tedesco et al., 2011). To investigate whether DLL4 and PDGF-BB-treated SCs acquired this property, we first performed a transwell assay on an H5V murine endothelial cell monolayer and measured the number of control or treated SCs that migrated through the endothelial layer after 6 h. Notably, treated cells had about a 3-fold increase in migration ability compared with untreated SCs (p < 0.01, N = 3; Figure 3E). Subcutaneously injected DLL4 and PDGF-BB-treated SCs transplanted into Matrigel plugs also appeared to migrate more toward CD31/PECAM+ host blood vessels, suggesting a response to chemotactic signals from the native endothelium (Figure S2, N = 2).

**Figure 4:**
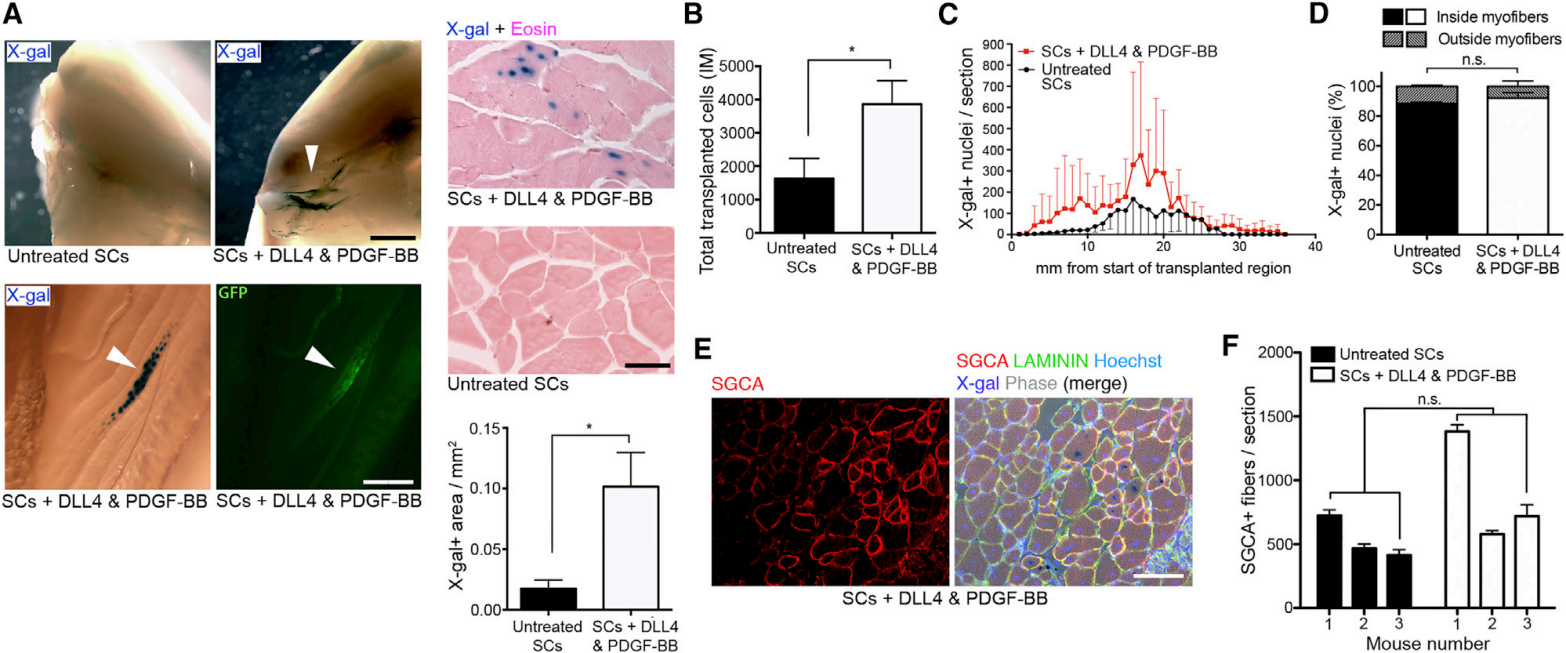
*In Vivo* Assessment of Migration and Engraftment of DLL4 and PDGF-BB-Treated SCs (A) *In vivo* analysis of limb muscles from *Sgca-null/scid/beige* mice intra-arterially transplanted with 10^6^ GFP+/β-GAL+ control or DLL4 and PDGF-BB-treated SCs after 3 weeks. Upper panel: exposure to X-gal revealed engraftment of β-GAL+ donor cells (blue; arrowhead) in the knee region and lower limb muscles of mice transplanted with DLL4 and PDGF-BB-treated SCs. Lower panel: assessment of X-gal in the gastrocnemius muscle of mice transplanted with treated SCs, revealing nuclear localization of β-GAL in a GFP+ myofiber (arrowheads). Graph quantifies the area of X-gal+ signal in muscles intra-arterially transplanted with treated or untreated SCs (lateral view of knee region) normalized to the volume of cell suspension delivered (N = 4). (B) Quantification of transplanted cells that engrafted intramuscularly injected TA muscles (N = 5). (C) Distribution across muscle length of X-gal+ nuclei per section of TA muscles transplanted with treated and untreated SCs (N = 5). (D) Quantification of the localization (i.e., inside or outside myofibers) of X-gal+, treated/untreated, donor-derived nuclei in transplanted muscles (N = 5). (E) Immunofluorescence panel showing a representative *Scga-null/scid/beige* muscle transplanted with 10^6^ GFP+/*nLacZ*+ DLL4 and PDGF-BB-treated SCs intramuscularly 3 weeks before explant. SGCA (red) and X-gal (violet): donor-derived fibers and nuclei, respectively. LAMININ (green) and Hoechst (blue): extracellular matrix and nuclei, respectively. (F) Quantification of the experiment in (E) showing the average number of donor-derived fibers from three mice. Data: means ± SEM. Statistical significance based on unpaired Welch's t test. ^∗^p < 0.05; n.s., not significant. Scale bars, 2 mm (A, left panel), 250 μm (A, middle panel), 50 μm (A, right panel), and 50 μm (E).

Proof-of-principle *in vivo* testing was then performed to determine whether improved migration through an endothelial monolayer *in vitro* resulted in SCs with the capacity to cross an actual blood vessel wall. To easily visualize cells, they were transduced with a lentivirus carrying a nuclear LacZ cDNA (*nLacZ*), which enables β-galactosidase (β-GAL)-mediated identification of donor cells with the X-gal reaction. A total of 10^6^ control or treated *GFP*+/*nLacZ*+ SCs were injected into the femoral arteries of dystrophic immunodeficient α-sarcoglycan-null/scid/beige mice (*Sgca*^*tm1Kcam*^*Prkdc*^*scid*^*Lyst*^*bg*^, *Sgca-null/scid/beige* hereafter; Tedesco et al., 2012) as reported previously (Gerli et al., 2014). This strain was chosen as it displays a more severe dystrophic phenotype compared with the *mdx* mouse and does not have revertant fibers. Prior to transplantation cells were stained with X-gal to determine the efficiency of transduction (84% X-gal+). Three weeks after transplantation, scattered GFP+/X-gal+ donor-derived nuclei were observed in muscles downstream of the injection site in limbs transplanted with DLL4 and PDGF-BB-treated SCs, with muscles receiving untreated SCs showing little to no signal (Figure 4A). The relatively low frequency of the event made it difficult to quantify with conventional methods (e.g., nuclei/section); however, the average X-gal+ area in the medial aspect of the hindlimb muscles increased from 0.02 mm^2^ to 0.1 mm^2^ (p = 0.028, N = 4, Figure 4A), suggesting an acquired ability of treated SCs to cross the blood vessel wall upon intra-arterial delivery. X-gal staining of filter organs (i.e., liver, spleen, kidneys, and lungs) showed no donor nuclei in mice receiving control or DLL4 and PDGF-BB-treated SCs intra-arterially (data not shown). These experiments show that DLL4 and PDGF-BB treatment induces perivascular properties to SCs such as endothelial branch stabilization, as well as improved migration *in vitro* and *in vivo*.

### DLL4 and PDGF-BB-Treated Mouse SCs Engraft Skeletal Muscle following Intramuscular Transplantation

To investigate if the increased migration resulted in improved *in vivo* engraftment while retaining their myogenic memory, 1 × 10^6^ GFP+/β-GAL+ SCs were transplanted intramuscularly into the tibialis anterior (TA) muscle of *Sgca-null/scid/beige* mice (N = 5, Figures 4B–4F). Three weeks after transplantation, explanted muscles were cryo-sectioned and co-stained for X-gal and α-sarcoglycan (SGCA) to detect donor-derived cells and their contribution to host tissues. This analysis revealed that the number of engrafted cells significantly increased with DLL4 and PDGF-BB treatment (p = 0.047, Figure 4B). Although TA muscles engrafted with treated SCs appeared to have more cells per section and a greater migration throughout the muscle than control SCs (Figure 4C), the percentage of X-gal+ nuclei located within myofibers (i.e., donor myonuclei) was unchanged with the treatment (Figure 4D). Finally, a similar number of SGCA+ myofibers was also observed (Figures 4E and 4F), indicating full differentiation capacity of treated SCs. In parallel, tumor formation assays based on subcutaneous injection of 2 × 10^6^ treated SCs were performed to assess safety of the signaling manipulation. After a minimum 2-month follow-up, no tumors were seen (N = 5 mice), indicating that the treatment did not result in oncogenic transformation of treated cells. These data show that, upon treatment with DLL4 and PDGF-BB, mouse SC-derived myoblasts have improved engraftment in host skeletal muscle than untreated cells, although with comparable contribution to myofiber repair/regeneration.

### Human SC-Derived Myoblasts Respond to DLL4 and PDGF-BB Stimulation

**Figure 5:**
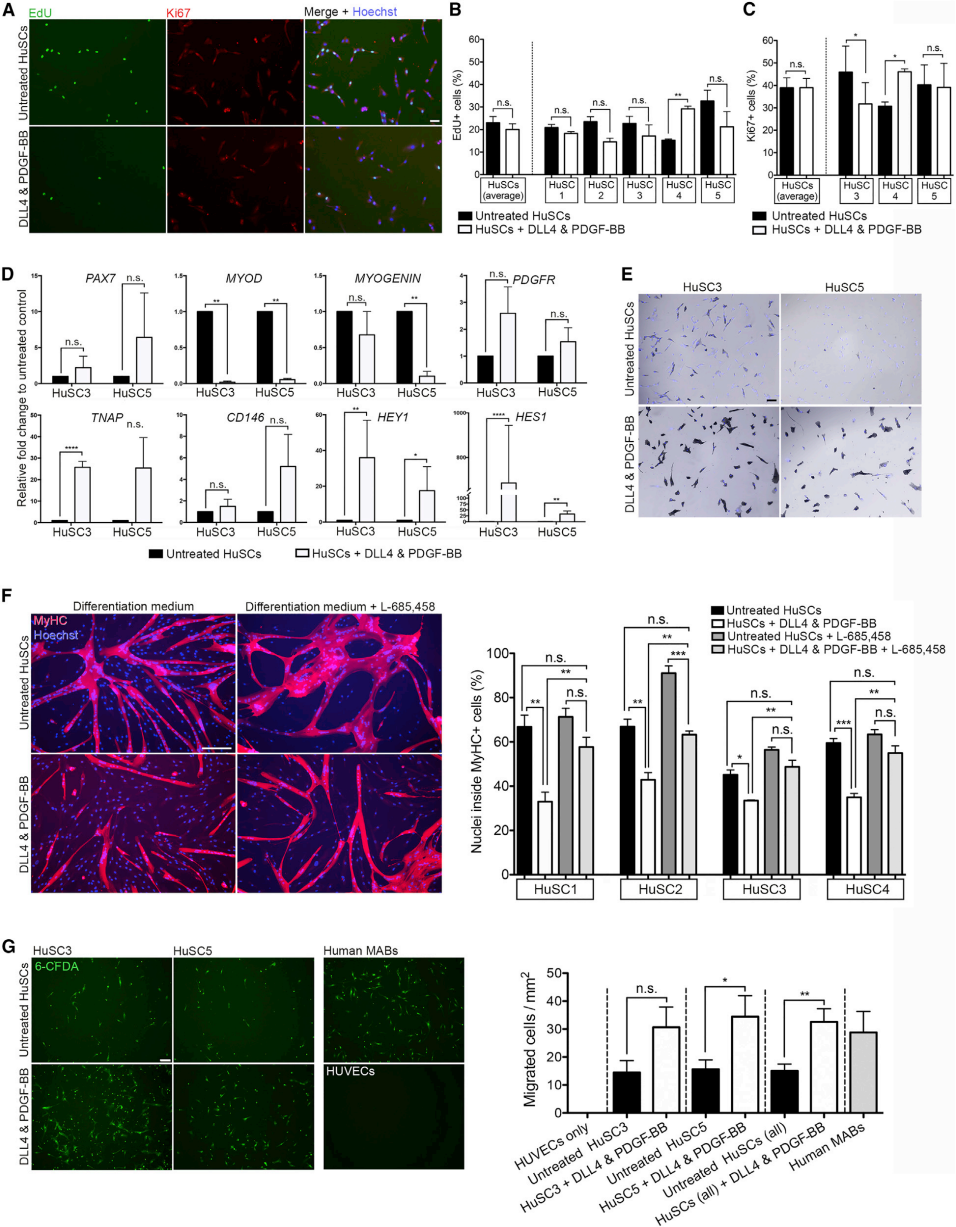
Effect of DLL4 and PDGF-BB Treatment on Human SC-Derived Myoblasts (A) Representative images of CD56+ FACS-purified human SC-derived myoblasts from two individuals (HuSC 3 and 5) expanded in control conditions (upper panel) or treated with DLL4 and PDGF-BB (lower panel) for 1 week, then pulsed for 2 h with EdU (green) and co-immunostained with Ki67 (red). (B) Quantification of the percentage of EdU+ cells (N = 5). (C) Quantification of Ki67+ cells (N = 3). (D) Real-time qPCR analysis of myogenic (*PAX7*, *MYOD*, and *MYOGENIN*), perivascular (*PDGFRB*, *TNAP*, and *CD146*) and Notch signaling (*HES1* and *HEY1*) genes in response to DLL4 and PDGF-BB treatment in HuSC 3 and 5 (n = 3 per line). Graphs show fold change to control conditions, statistical significance based on ΔCt. (E) Phase contrast images of AP enzymatic activity (violet) and Hoechst (blue) in untreated and treated HuSCs. (F) Differentiation of HuSCs in response to DLL4 and PDGF-BB treatment and Notch inhibition. Cells expanded in control or treatment conditions for 1 week and induced to differentiate for 4 days in the presence or absence of L-685,458. Pictures representatives from HuSC1, and graphs quantify the percentage of MyHC+ cells (N = 4). (G) Representative images of the lower chambers of transwell assays. Untreated/treated HuSCs and human muscle pericyte-derived mesoangioblasts (MABs; technical control) were seeded for 8 h on HUVECs. Right graph: number of 6-CFDA+ migrated cell/mm^2^ in each condition. N = 3; means ± SEM. Statistical significance based on paired Student's t test (B, C and cell-line-specific data in G), unpaired Student's t test (D and pooled data in G) or one-way ANOVA with Tukey's multiple comparison (F). ^∗^p < 0.05, ^∗∗^p ≤ 0.01, ^∗∗∗^p ≤ 0.001, ^∗∗∗∗^p ≤ 0.0001; n.s., not significant. Scale bars, 50 μm (A), 75 μm (F), and 100 μm (E and G).

Translation of this strategy into future pre-clinical studies requires evidence that the mechanism is conserved also in human SC-derived myoblasts (hereafter referred to as HuSCs). To this aim, primary human muscle biopsy-derived SCs from five individuals (HuSCs 1–5) were isolated and purified by fluorescence-activated cell sorting (FACS) for CD56 positivity, which marks HuSCs (Boldrin et al., 2010). Purified SCs were expanded and treated with human DLL4 and PDGF-BB for 7 days or expanded in control conditions. Proliferation analyses (Figures 5A–5C) revealed that, unlike murine SCs, the percentage of EdU+ cells was unaffected by DLL4 and PDGF-BB treatment (Figure 5B). The exception to this was donor no. 4, where a significant increase was observed (p = 0.002). The percentage of Ki67+ cells was assessed in three HuSC preparations to confirm EdU analyses, which, although variable between preparations, was unaffected when results were averaged (Figure 5C).

Analysis of transcriptional changes elicited by DLL4 and PDGF-BB treatment showed activation of Notch signaling via increased *HEY1* (HuSC3 p = 0.006, HuSC5 p = 0.028) and *HES1* (HuSC3 p > 0.0001, HUSC5 p = 0.003), although differences in expression levels were observed between the preparations assessed (Figure 5D). A trend in the increase of *PDGFRB* transcripts was also observed, although not statistically significant. As with murine SCs, myogenic markers *MYOD* (HuSC3 p = 0.006, HuSC5 p = 0.008) and *MYOGENIN* (HuSC5 p = 0.007) were decreased, whereas the pericyte marker *TNAP* was increased (p < 0.0001). This correlated with an increase in AP enzymatic activity (Figure 5E). *PAX7* expression did not increase significantly with treatment, although all samples expressed very low baseline levels, likely due to cell expansion in culture, or distinct regulation of this gene in the human compared with mouse (Figure 5D).

We then tested HuSC differentiation in response to DLL4 and PDGF-BB treatment. To determine whether any changes were due to Notch, cells were differentiated in low mitogen medium for 4 days in the presence or absence of γ-secretase inhibitor (Figure 5F). In all preparations assessed, DLL4 and PDGF-BB treatment reduced myogenic differentiation, which was rescued by inhibiting Notch signaling (Figure 5F).

To assess whether transcriptional changes induced by DLL4 and PDGF-BB treatment had functional consequences in HuSCs, transmigration ability was assessed via transwell assay on a monolayer of HUVECs (Figure 5G, N = 3). After 8 h, treated cells showed an average 2-fold overall increase in migration capacity, from 15.02 to 32.54 cells/mm^2^ (p = 0.008), although some variability was observed among the lines assessed (e.g., p = 0.144 for HuSC3 and p = 0.046 for HuSC5). Taken together, these results indicate that DLL4 and PDGF-BB induce cell fate changes and improve migration also in HuSCs, laying the foundation for further studies on this strategy for human muscle cell therapy.

## Discussion

In this work, we hypothesized that cell fate determinants involved in smooth and skeletal muscle lineage determination in the embryo may exert a similar effect also on adult SCs, and that their modulation could be exploited to improve skeletal muscle cell therapies. Direct lineage reprogramming (also known as trans-differentiation) is the conversion of one functional cell type to another fate without an intermediate pluripotent step (Xu et al., 2015). Here, we show that a phenomenon resembling reversible lineage reprogramming takes place in adult SCs in response to DLL4 and PDGF-BB stimulation. Adult SC-derived myoblasts treated with DLL4 and PDGF-BB increase expression of perivascular genes. At variance with their embryonic counterpart (Cappellari et al., 2013), adult SCs acquired also an expression profile suggestive of self-renewing SCs (i.e., high PAX7 and low MYOD). However, the co-expression of SC and perivascular markers indicates that the treatment does not trigger a complete trans-differentiation, but rather a stimulus-dependent reprogramming into a hybrid cell type. Our isolation procedures (FACS purification using PAX7-GFP for murine cells and CD56 for their human counterpart) minimize the possibility that the observations are due to contaminating cells.

Similar to bona fide pericytes, treated SCs become associated with endothelial networks (Armulik et al., 2005). Notably, transwell assays and *in vivo* experiments showed improved ability of treated SCs to cross endothelial barriers, suggesting acquisition of a similar machinery that pericyte-derived mesoangioblasts might use to cross the blood vessel wall (Giannotta et al., 2014).

Manipulating Notch signaling affects SC differentiation and transplantation ability. Recently, it was shown that while Jagged1, DLL1, or DLL4 alone can maintain a stem-like phenotype in SCs grown *ex vivo*, this does not correlate with an increased efficiency to generate myofibers upon transplantation (Sakai et al., 2017). Interestingly, our results indicate that, when combined with PDGF-BB, DLL4 induces perivascular features in SCs and increases their engraftment into host skeletal muscle, albeit with a similar contribution to myofibers. We hypothesize that this might be due to increased migration throughout the host muscle resulting in fusion of more donor cells with the same myofibers. Hence, this migration-enhancing strategy preserves the overall SC engraftment and differentiation potential without altering their safety profile, as shown by lack of oncogenic transformation in transplantation experiments and tumorigenesis assays.

It is thought-provoking to speculate that satellite and perivascular cells might retain bidirectional plasticity in adulthood due to a residual developmental memory from a common precursor. It has been shown that skeletal and vascular smooth muscle cells have a common PAX3+ progenitor in the dermomyotome (Esner et al., 2006, Goupille et al., 2011, Lagha et al., 2009). At later stages of development, it has been shown that embryonic myoblasts and skeletal muscle pericytes maintain the ability to trans-differentiate toward a smooth or skeletal muscle fate, in response to DLL4, PDGF, and Noggin signaling, respectively (Cappellari and Cossu, 2013). Although experiments in mice challenge the model of endogenous pericytes as tissue-resident progenitors (Guimaraes-Camboa et al., 2017), there is evidence that human skeletal myogenic progenitors are associated with blood vessel walls *in vivo* in perivascular locations typical of mural cells/pericytes or adventitial cells of muscular veins (Dellavalle et al., 2007, Sacchetti et al., 2016). This population has been hypothesized to represent either a subset of myogenic progenitors recruited to separate niches based on proximity (i.e., satellite vs vascular niche), or, alternatively, to be a class of progenitors upstream of SCs. Both models would fit with our data, with DLL4 and PDGF-BB treatment either mimicking *in vitro* what hypothesized by the first model or bringing the cells back to their primitive nature, as per the second model. Although *ex vivo* culture is a key procedure used in this study, previous *in vivo* evidence in the developing embryo underpins this skeletal muscle-vascular crosstalk in adult SCs. Specifically, we demonstrated that SCs are responsive to DLL4 and PDGF-BB treatment irrespective of mouse strain (CD57BL/6 and CD1), isolation procedure (pre-plating or PAX7-GFP sorting), tissue culture substrate, timing of treatment (i.e., immediately upon isolation or following expansion) or species (mouse and human).

Future applications of this strategy include its extension to pluripotent cell-derived myogenic cells, which we and others are developing as a promising tool for regenerative medicine (Loperfido et al., 2015). Taken together, our data indicate that SC plasticity toward the perivascular lineage is conserved in adulthood and opens the possibility to exploit this phenomenon to generate clinically relevant stem cell populations for future cell therapies of muscle disorders.

## Experimental Procedures

### Animals

Mouse strains used in this study: *Tg:Pax7-nGFP* F1:C57BL/6:DBA2 expressing nuclear-localized EGFP in *Pax7*-expressing cells (Sambasivan et al., 2009); *Tg:TN-AP-CreERT2* (Dellavalle et al., 2011); Gt(ROSA)26Sor^tm14(CAG-tdTomato)Hze^, a *Td:Tomato* reporter in *Cre*-expressing cells (Madisen et al., 2010) hereafter referred to as *Td:Tomato*; *R2*6R*-EYFP*, a reporter expressing cytoplasmic YFP in *Cre*-expressing cells (Srinivas et al., 2001); *Tg:CAG-EGFP* mice expressing ubiquitous GFP (Okabe et al., 1997); CD1 and *Sgca-null/scid/beige* mice (*Sgca*^*tm1Kcam*^*Prkdc*^*scid*^*Lyst*^*bg*^; Tedesco et al., 2012). Housing of all mice (except *Tg:Pax7-nGFP*) and all experimental procedures were performed in accordance with British law under the provisions of the Animals (Scientific Procedures) Act 1986, as approved by the University College London Ethical Review Process committees and under UK Home Office Project Licence 70/8566. Surgery was performed under isoflurane anesthesia and every effort made to minimize pain and suffering, including use of analgesia. *Tg:Pax7-nGFP* mice were bred at the Institut Pasteur (Paris, France), in compliance with European legislation and under the approval of the institutional ethical committee.

### Cell Isolation, Culture and Assays

Primary murine SCs and pericytes were isolated from dissected adult skeletal muscles and cultured as detailed in Supplemental Experimental Procedures. HuSCs were obtained from the MRC Neuromuscular Center Biobank (UCL, London, UK; Research Ethics Committee reference no. 06/Q0406/33). Human cell work was conducted under the approval of the NHS Health Research Authority Research Ethics Committee reference no. 13/LO/1826; IRAS project ID no. 141100. Additional details, including full information on cell culture, purification and protocols used in morphological, proliferation, differentiation, network formation, migration, and transplantation assays are available in Supplemental Experimental Procedures.

### DLL4 and PDGF-BB Treatment

Murine recombinant DLL4 (R&D Systems; 1389-D4-050) was resuspended to a concentration 10 μg/mL in sterile PBS containing 1% wt/vol BSA. Flasks were coated with DLL4 at 37°C for 45 min before seeding cells. Cultures were supplemented daily with 50 ng/mL of recombinant PDGF-BB (Sigma; P4056) resuspended in 0.1% BSA/4 mM HCl/PBS. For human myoblast treatment, recombinant human DLL4 (R&D Systems; 1506- D4-050) and human PDGF-BB (R&D Systems; 220-BB-050) were dissolved and treated as murine proteins to final concentrations of 10 μg/mL and 100 ng/mL, respectively. Cells were assessed after at least 7 days of treatment, by which time they had been passaged at least once on fresh DLL4-coated plates.

### Immunofluorescence and Enzymatic Staining

Cells and tissue sections were fixed in 4% paraformaldehyde, permeabilized using 0.1% Triton and 1% BSA PBS and blocked in 10% donkey or goat serum in PBS for 60 min. Samples were then incubated overnight at 4°C with the following primary antibodies: mouse anti-MyHC (DSHB MF20, 1:10), mouse anti-CD31/PECAM (DSHB P2B1, 1:2), mouse anti-PAX7 (DSHB, 1:1), rabbit anti-Ki67 (Leica NCL-Ki67p, 1:1,000), mouse anti-α-sarcoglycan (anti-SGCA, Novocastra NCL-a-SARC, 1:150 and Abcam AB49451, 1:150), rabbit anti-SGCA (Abcam EPR14773, 1:1,000 on unfixed sections) rabbit anti-LAMININ (Abcam AB11575, 1:300), rabbit anti-MYOD (Santa Cruz Biotechnology M-318, 1:300). Samples were then incubated with fluorescently-conjugated donkey or goat secondary antibodies (Molecular Probes, Alexa Fluor series, 1:500). Hoechst 33342 (Fluka B2261) was used to counterstain nuclei. AP and X-gal staining were performed by incubating cells at room temperature or 37°C, respectively, until a colorimetric change was observed (Roche NBT/BCIP-11681451001 and Sigma 3117073001).

### Gene Expression Analyses

RNA extraction was performed with RNeasy kits (QIAGEN), yield and purity assessed with a Nanodrop spectrophotometer and reverse transcribed into cDNA with the Improm-II Reverse Transcription System kit (Promega; A3800). Relative gene expression values were measured by real-time qPCR using the SYBR Green Real-Time Master Mix (Promega; A600A) on a Bio-Rad CFX96 machine. Real-time qPCRs were performed in triplicate on samples from at least three independent experiments. *Gapdh* and *RPLPO* were used as housekeeping genes for murine and human experiments, respectively. Data presented as means ± SEM of the fold change, normalized to control SCs. Significance was assessed on the delta Ct values using Student's t test assuming two-tailed distribution and equal variances. Sequences of primers available in Supplemental Experimental Procedures.

### Statistics

Statistical testing was performed using Microsoft Excel and GraphPad Prism software. N values and experimental replicates are detailed in figure legends. “N” refers to independent experiments or animals (mice or litters); “n” refers to technical replicates. Graphs display means ± SEM unless otherwise stated. Statistical testing was based on t tests or one-way ANOVA with specific *post-hoc* comparison tests as detailed in figure legends.

## Author Contributions

Conceptualization, F.S.T., M.F.M.G., and L.A.M.; Methodology, M.F.M.G., L.A.M., S.B., E.U., M.R., C.C., I.L., H.S., and F.S.T.; Investigation, M.F.M.G., L.A.M., S.B., G.F., and F.S.T.; Writing – Original Draft, M.F.M.G., L.A.M., and F.S.T.; Writing – Review & Editing, M.F.M.G., L.A.M., G.C., S.T., and F.S.T.; Funding Acquisition, F.S.T.; Resources, G.C., S.T., P.A., P.D.C., and F.S.T.; Supervision and Coordination, F.S.T.; M.F.M.G. and L.A.M. contributed equally; S.B., G.F., and E.U. contributed equally.

## Supplemental Information

Document S1. Supplemental Experimental Procedures and Figures S1 and S2

Document S2. Article plus Supplemental Information
