# Supplementary material for: Combined Notch and PDGF Signaling Enhances Migration and Expression of Stem Cell Markers while Inducing Perivascular Cell Features in Muscle Satellite Cells
Source: Stem Cell Reports. 2019 Feb 7;12(3):461–73. doi: 10.1016/j.stemcr.2019.01.007 (PMC6409426; doi:10.1016/j.stemcr.2019.01.007)
Supplement: Document S1. Supplemental Experimental Procedures and Figures S1 and S2 [file mmc1.pdf]

Stem Cell Reports, Volume *12*

Supplemental Information

Combined Notch and PDGF Signaling Enhances Migration and Expression of Stem Cell Markers while Inducing Perivascular Cell Features in Muscle Satellite Cells

Mattia Francesco Maria Gerli, Louise Anne Moyle, Sara Benedetti, Giulia Ferrari, Ekin Ucuncu, Martina Ragazzi, Chrystalla Constantinou, Irene Louca, Hiroshi Sakai, Pierpaolo Ala, Paolo De Coppi, Shahragim Tajbakhsh, Giulio Cossu, and Francesco Saverio Tedesco

SUPPLEMENTAL INFORMATION

Combined Notch and PDGF Signaling Enhances Migration and Expression of Stem Cell Markers while Inducing Perivascular Cell Features in Muscle Satellite Cells

Mattia Francesco Maria Gerli, Louise Anne Moyle, Sara Benedetti, Giulia Ferrari, Ekin Ucuncu, Martina Ragazzi, Chrystalla Constantinou, Irene Louca, Hiroshi Sakai, Pierpaolo Ala, Paolo De Coppi, Shahragim Tajbakhsh, Giulio Cossu and Francesco Saverio Tedesco

INVENTORY:

- **SUPPLEMENTAL FIGURES**
  - **Figure S1.** Additional Analyses of DLL4 & PDGF-BB Treated Adult Murine SCs, Related to Figure 2.
  - **Figure S2.** Migration of Treated SCs within Subcutaneous Matrigel Plugs, Related to Figure 3.
- **SUPPLEMENTAL EXPERIMENTAL PROCEDURES**
  - Cell isolation, culture and differentiation
  - Morphological and proliferation analyses
  - Network formation and migration assays
  - Cell transplantation
  - Sequences of primers used for RT-qPCR
- **SUPPLEMENTAL REFERENCES**

SUPPLEMENTAL FIGURES

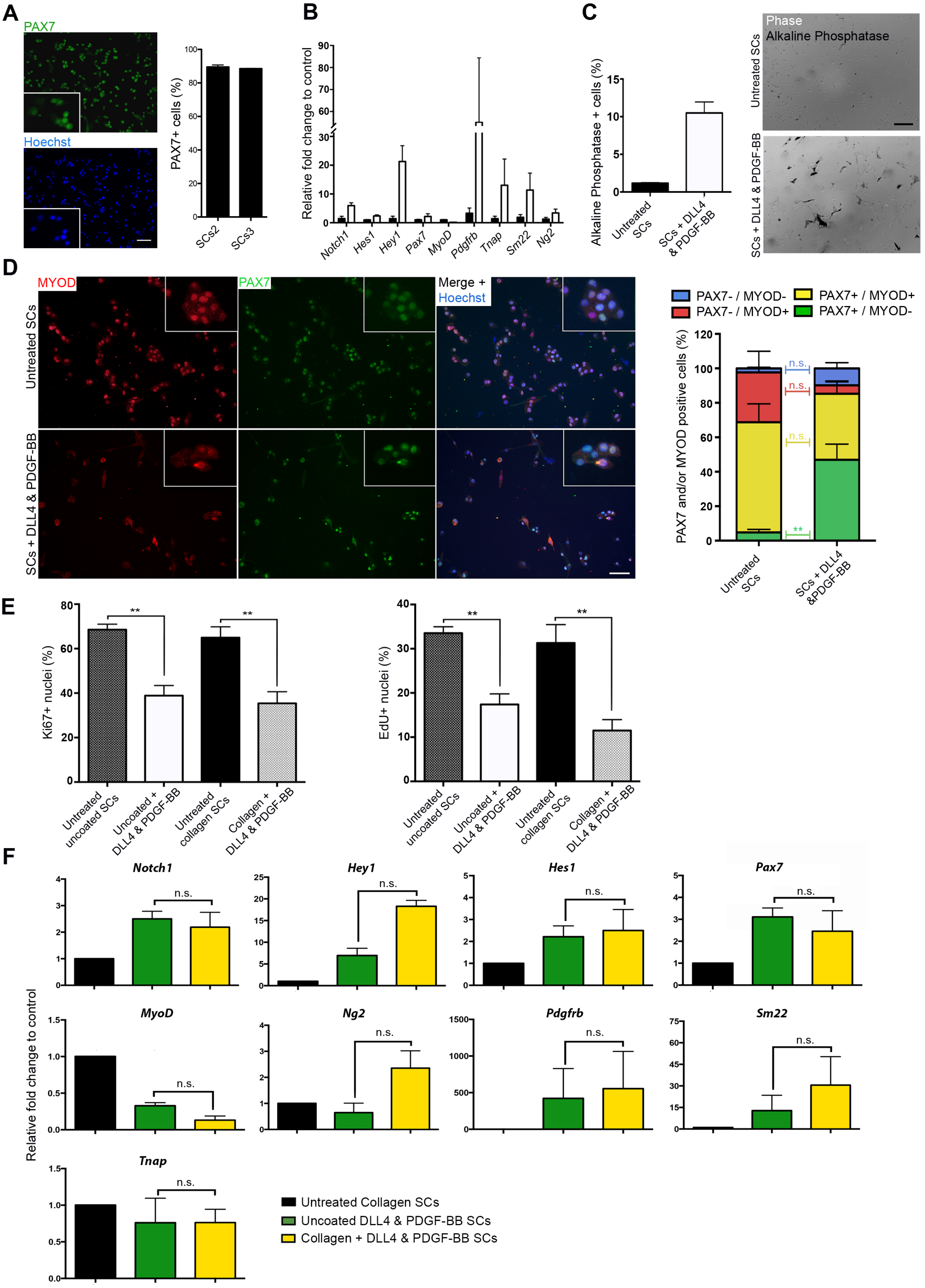

Figure S1. Additional analyses of DLL4 & PDGF-BB-treated adult murine SCs; related to Figure 2. (A). Left: representative immunofluorescence panel showing PAX7 (green) staining on murine SCs isolated with the pre-plating technique and cultured for 2 passages after isolation before staining; Hoechst (blue): nuclei. Right: graph quantifying the percentage of PAX7+ cells in two SC preparations (N = 3, a minimum of 500 nuclei were counted per preparations). **(B)** RT-qPCR expression analysis of SC genes (*Pax7, MyoD*), smooth muscle genes (*Tnap, Ng2,* and *Sm22*) and Notch and PDGF signaling effectors (*Notch1, Hes1*, *Hey1 and Pdgfrb*) in control (black bars) and DLL4 & PDGF-BB-treated (white bars) SCs from CD1 mice. Graphs show fold change to control, statistical significance based on ΔCt (n = 3). **(C)** Alkaline phosphatase activity in untreated and treated SCs (n = 3). **(D)** Immunofluorescence images of SCs expanded for 2 weeks prior to DLL4 & PDGF-BB treatment and co-immunostained with antibodies against PAX7 (green) and MYOD (red). The relative percentages of PAX7+/MYOD-, PAX7+/MYOD +, PAX7-/MYOD+ and PAX7-/MYOD- cells were quantified (N = 4). **(E)** Treatment of PAX7-GFP+ SCs on uncoated or collagen-coated dishes (N = 3). Left graph shows the percentage of Ki67+ cells decreasing irrespective of coating in response to treatment: from 64.94% in untreated collagen to 35.40% in collagen + DLL4 & PDGF-BB (p = 0.023), and from 70.79 % in untreated collagen to 42.50% in uncoated + DLL4 & PDGF-BB ($p = 0.044$). Right graph shows the percentage of EdU+ cells decreasing irrespective of coating in response to treatment: from 31.30% in untreated collagen to 11.49% in collagen + DLL4 & PDGF-BB ($p = 0.006$), and from 34.20% in untreated collagen to 18.60% in uncoated + DLL4 & PDGF-BB ($p = 0.002$). **(F)** RT-qPCR expression analysis of the same genes shown in (B) in response to DLL4 & PDGF-BB treatment on collagen-coated or uncoated dishes (no difference observed). Graphs presented as mean ± SEM and statistical significance based on two-way ANOVA with Bonferroni's (C) or Tukey's (D, F) multiple comparison, or a paired student's *t*-test (E). *$p < 0.05$, **$p < 0.01$. Scale bars 100 μm (B) and 50 μm (A, C).

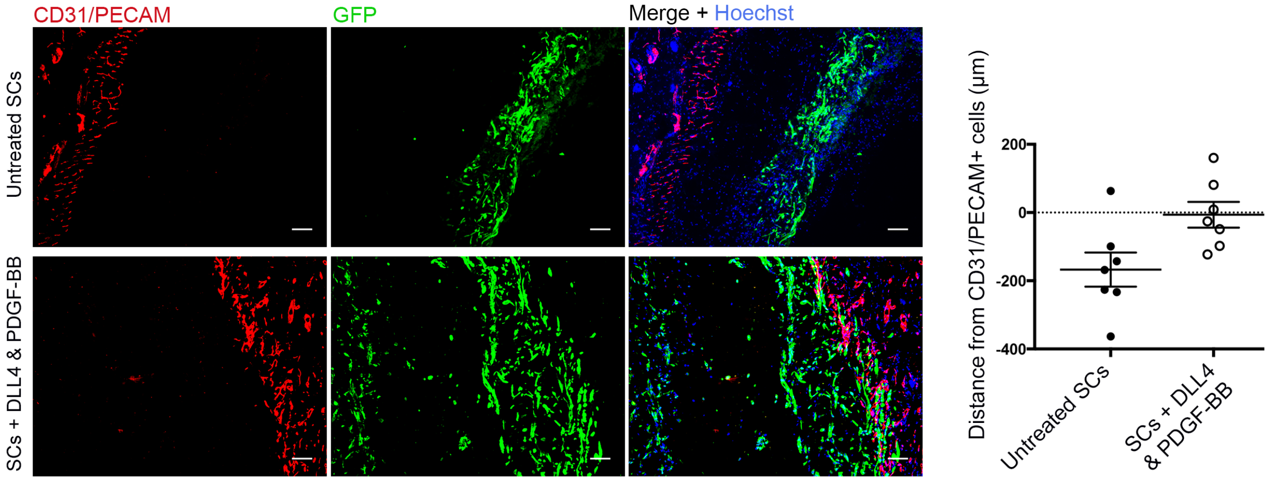

Figure S2. Migration of treated SCs within subcutaneous Matrigel plugs; related to Figure 3. Representative images of *in vivo* Matrigel plugs from mice injected with untreated (top panel) or DLL4 & PDGF-BB-treated SCs (lower panel) immunostained for PECAM/CD31 (red), GFP (green) and Hoechst nuclear stain (blue). The distance between GFP+ SCs and CD31/PECAM+ cells of the host vasculature was calculated (negative values = distance away from nearest blood vessel, 0 = in contact, positive values = distance inside the host vasculature bed) (N = 2). Scale bars 100 µm.

SUPPLEMENTAL EXPERIMENTAL PROCEDURES

Cell isolation, culture and differentiation

Primary SCs were isolated from dissected adult muscles by mechanical fragmentation and digested twice in 0.1% Collagenase D (Roche) + 0.24U/ml Dispase (Gibco) + 0.1 mg/ml DNase (Roche), diluted in DMEM (Sigma-Aldrich) for 20 minutes at 37°C in agitation. The digestion product was filtered through a 40 µm cell strainer (Thermo Fisher) and enzyme activity inhibited with 20% fetal bovine serum (FBS; Life Technologies). The mononuclear cell suspension was washed twice in phosphate buffered saline (PBS) and pre-plated on cell culture dishes at 37°C 5% CO_2 for 30 minutes to remove fibroblast contamination and enrich the floating fraction of less adherent SCs. The supernatant was collected and transferred to dishes coated with collagen (C8919 from calf skin Sigma-Aldrich) at a density

of 1.2 x 10^3 cells/cm^2. Mononuclear cell suspensions from 6 to 8-week old female *Tg:Pax7-nGFP* mice were FACS-purified in PBS + 2% FBS + 2mM EDTA (Invitrogen) on a MoFlo XDP cell sorter (Beckman Coulter).

Primary murine myogenic pericytes were isolated based on TN-AP expression as previously reported (Dellavalle et al., 2011). Briefly, *TN-AP-CreERT2* mice were crossed with Td: Tomato or $R26R^{EYFP}$ lines and resulting litters subcutaneously injected daily with 0.3 mg tamoxifen (Sigma-Aldrich) from P6-8 to induce Cre-recombination. Muscles were harvested at P19-21, digested as described above and pericytes FACS-purified on a FACS Aria III (BD Biosciences) or MoFlo XDP (Beckman Coulter).

Primary murine SCs and muscle pericytes were cultured at low density on collagen-coated dishes at 37°C, 3% O_2 and 5% CO_2 in primary murine growth medium: DMEM-F/12 (Gibco, USA) supplemented with 20% FBS , 10% horse serum (HS)(Euroclone), 1% non-essential amino acids (NEAA; Sigma-Aldrich), 1 mM sodium-pyruvate (Sigma-Aldrich), 1% insulin-transferrin-selenium (Sigma-Aldrich), 2 mM glutamine (Sigma-Aldrich), 50 μM β-mercaptoethanol (Gibco), 1% penicillin-streptomycin (P/S; Sigma-Aldrich), 50 μg/ml gentamycin (Gibco) and 5 ng/ml bFGF (Gibco).

Primary human SC-derived myoblasts (HuSCs) were obtained from the MRC Neuromuscular Centre Biobank and tissue sampling was conducted under the approval of the NHS Health Research Authority Research Ethics Committee (NHS HRA REC) reference no. 06/Q0406/33. Human cell work conducted under NHS HRA REC no. 13/LO/1826, IRAS project ID no. 141100. To increase purity, HuSCs were isolated by FACS for CD56+ (Biolegend; CD56-FITC 304604). Cells were cultured at low density in MegaCell DMEM medium (Sigma-Aldrich) supplemented with 5% FBS, 2 mM glutamine, 1% NEAA, 1% P/S, 50 μM β-mercaptoethanol and 5ng/ml bFGF. Primary human pericyte-derived mesoangioblasts (MABs) were the 27XY preparation from (Bonfanti et al., 2015), which were cultured in the same MegaCell-based medium as HuSCs.

C2C12 murine myoblasts (used to produce conditioned-medium) (Yaffe and Saxel, 1977) and H5V murine endothelial cells (Garlanda et al., 1994) were cultured at 37°C, 3% O_2, 5% CO_2 in DMEM supplemented with 10% FBS, 2 mM glutamine and 1% P/S. Primary human umbilical vein endothelial cells (HUVECs; Lonza) were maintained at 37°C, 5% CO_2 in EGM1 (Lonza) on 1% gelatin-coated flasks (Sigma-Aldrich). HUVECs were kept below 70% confluence and used until passage 6.

For myogenic differentiation assays, cells were seeded at a high density (1.2 x 10^4 cells/cm^2) on collagen-coated dishes and grown until confluence, then switched to differentiation medium (DMEM supplemented with 2% HS + 1% P/S), replaced every other day for 4 days before fixation. To block Notch signaling, samples were incubated with 660 ng/ml L-685,458 (γ-secretase inhibitor; Sigma-Aldrich) 24 hours prior to the medium-switch and added to each subsequent medium change.

Morphological and proliferation analyses

Phase contrast images of proliferating cells were analyzed to measure the cell circularity ratio (1 = circle; 0 = line). The cell contours were drawn and quantified using Image J software. A minimum of 500 cells were analyzed from 3 random fields per condition, and each experiment was performed with at least 3 independent repeats.

To measure proliferation rate, cells were pulsed for 2 hours with 10 µM 5- Ethynyl-2'-deoxyuridine (EdU) before fixation and processing following manufacturer's instructions (Click-iT® EdU Alexa Fluor® Imaging Kit, Life Technologies). To generate growth curves cells were cultured for three weeks, trypsinized every other day and counted using Glasstic slides (Kova; 87144).

Network formation and migration assays

For endothelial network formation assays, 24 multi-well dishes were coated for 30 minutes

at 37°C with growth factor-reduced Matrigel (Corning) diluted 1:1 in EGM1 medium (Lonza). 5 x 10^4 HUVECs/cm^2 were then seeded on top of the Matrigel-coated dishes, followed after 30 minutes by 10^4 GFP+ SCs or pericytes from *Tg:TN-AP-CreERT2:R26R-EYFP* mice (ratio of 1:10 to HUVECs), together with 10 ng/ml of recombinant human vascular endothelial growth factor (VEGF, R&D Systems). After 24 hours the number of network branches / high-power field (HPF; 10X lens = 1.5 mm^2) was quantified with an inverted fluorescence microscope (Leica). For quantification, 2-5 random pictures per well were taken and the data displayed as numbers of network branches/mm^2. Four independent experiments were performed by 4 independent operators, with each sample repeated in duplicate in at least 3 experiments. Association of fluorescent cells with the HUVEC network was also monitored over time up to 120 hours post-seeding.

For murine cell migration experiments, H5V endothelial cells were grown to confluence on 8 µm porous cell culture membranes (BD Falcon 353093) coated with 1% gelatin for 1 hour at 37°C. To assess trans-endothelial migration, 1 x 10^4 GFP+ SCs, *TN-AP:YFP+* primary pericytes or C57 *GFP+/nLacZ+* mesoangioblasts were seeded in the upper transwell chamber in serum-free medium. The lower chamber was filled with 50% growth medium + 50% C2C12 myotube-conditioned medium. After 6 hours, membranes were fixed for 5 minutes in 4% PFA and cells from the upper side of the membrane removed using a cell scraper. The average number of migrated cells was quantified from at least five 10X fields (1.5 mm^2). Transwell assays with human cells were performed as previously described (Giannotta et al., 2014). 2 x 10^5 HUVECs were seeded for 72 hours. Human SCs and mesoangioblasts were dissociated with TrypLE and labelled with 0.7 µM 6-carboxyfluorescin dictate (both ThermoFisher Scientific) for 30 minutes at 37°C and 5 x 10^4 cells seeded per membrane for 8 hours before processing and quantifying as described above.

Cell transplantation

Three to five-month-old *sgca-null/scid/beige* mice were used for transplantation experiments, which were performed as previously described (Gerli et al., 2014). Briefly, SCs from *Tg:CAG-EGFP* mice (Okabe et al., 1997) were transduced with a lentivirus encoding nuclear β-galactosidase (*nLacZ*) (Tedesco et al., 2011) and then expanded. For intramuscular injections, cells were trypsinized and resuspended in Ca^{2+}/Mg^{2+}-free PBS to a concentration of 1 x 10^6 cells/30 µl and injected with a 30G needle into tibialis anterior muscles (N = 5/condition). Two muscles were sham injected (PBS). For intra-arterial delivery, cells were filtered through a 40 µm strainer, re-suspended at a concentration of 1 x 10^6 cells/50 µl, and injected into femoral arteries using a 30G needle (N = 7/condition). Contralateral limbs were injected with control SCs and 2 limbs were sham injected (PBS). Muscles were harvested after 23 days and assessed for the presence of donor-derived cells, either as whole mounts or flash-frozen in isopentane-cooled in liquid nitrogen, then serially cryosectioned using a Leica CM 1850 UV cryostat. Analysis of transplantation was performed blinded, and intra-arterial transplantations were normalized to the volume of cells injected.

Tumorigenic assays were performed as previously reported (Tedesco et al., 2012). Briefly, 2 x 10^6 cells were harvested, re-suspended in 100µl of PBS and injected subcutaneously in immunodeficient scid/beige mice (N = 5). Animals were observed bi-weekly and euthanized after 2 months to confirm the absence of masses.

For subcutaneous plugs, cells were mixed 1:1 with HUVECs in Matrigel supplemented with 10 ng/ml VEGF (Sigma-Aldrich) and slowly injected subcutaneously into two 3-month-old nude mice using refrigerated syringes. Animals were anesthetized for 15 minutes to facilitate Matrigel polymerization. After 2 weeks, plugs were harvested, frozen in liquid nitrogen-cooled isopentane and cryosectioned.

Sequences of primers used for RT-qPCR

Name	Forward sequence 5'-3'	Reverse Sequence 5'-3'
Gapdh	TTCACCACCATGGAGAAGGC	GGCATGGACTGTGGTCATGA
Notch1	TGGACGCCGCTGTGAGTCA	TGGGCCCGAGATGCATGTA
Hes1	ACACCGGACAAACCAAAGAC	AATGCCGGGAGCTATCTTTC
Hey1	CACCTGAAAATGCTGCACAC	ATGCTCAGATAACGGGCAAC
Pax7	ATGTTCAGCTGGGAAATCCGGG	TCCCGAACTTGATTCTGAGCACTCG
MyoD	GCCCGCGCTCCAACTGCTCTGAT	GCCCGCGCTCCAACTGCTCTGAT
Pdgfrb	GCTCACGGTCTGAGCCATTC	GCTCGGACATTAAGGCTTGCT
Sm22	CCAACAAGGGTCCATCCTACG	ATCTGGGCGGCCTACATCA
Ng2	ACAAGCGTGGCAACTTTATC	ATAGACCTCTTCTTCATATT
Tnap	GTGGATACACCCCCCCGGGGC	GGTCAAGGTGGCCCCAATGCA
RPLPO	TCTACAACCCTGAAGTGCTTGAT	CAATCTGCAGACAGACACTGG
PAX7	CAAACACAGCATCGACGG	CTTCAGTGGGAGGTCAGGTT
MYOD	CACGTCGAGCAATCCAAACC	TGTAGTCCATCATGCCGTCG
MYOD	AATAAGAGTTGCTTTGCCAG	GTACAAATTCCCTGTAGCAC
MYOGENIN	CCAGGGGTGCCCAGCGAATG	AGCCGTGAGCAGATGATCCCC
PDGFRB	AGCTGTTACCCACTCTGGGA	TGGTGTCCTTGCTGCTGATG
TNAP	TGTGGGGTGAAGGCCAATG	GTGGTGGTCACAATGCCCA
CD146	GGAAGCAGGAGATCACGCTAC	GATTCGGGGCTAATGCCTCA
HEY1	AGGTTACTTTGACGCGCACG	ACCAGTCGAACTCGAAGCG
HES1	AGAAAGATAGCTCGCGGCA	TACTTCCCCAGCACACTTGG

SUPPLEMENTAL REFERENCES

Bonfanti, C., Rossi, G., Tedesco, F.S., Giannotta, M., Benedetti, S., Tonlorenzi, R., Antonini, S., Marazzi, G., Dejana, E., Sassoon, D., *et al.* (2015). PW1/Peg3 expression regulates key properties that determine mesoangioblast stem cell competence. Nat Commun *6*, 6364.

Garlanda, C., Parravicini, C., Sironi, M., De Rossi, M., Wainstok de Calmanovici, R., Carozzi, F., Bussolino, F., Colotta, F., Mantovani, A., and Vecchi, A. (1994). Progressive growth in immunodeficient mice and host cell recruitment by mouse endothelial cells transformed by polyoma middle-sized T antigen: implications for the pathogenesis of opportunistic vascular tumors. Proc Natl Acad Sci U S A *91*, 7291-7295.

Yaffe, D., and Saxel, O. (1977). Serial passaging and differentiation of myogenic cells isolated from dystrophic mouse muscle. Nature *270*, 725-727.
