# Supplementary material for: Combined Notch and PDGF Signaling Enhances Migration and Expression of Stem Cell Markers while Inducing Perivascular Cell Features in Muscle Satellite Cells
Source: Stem Cell Reports. 2019 Feb 7;12(3):461–73. doi: 10.1016/j.stemcr.2019.01.007 (PMC6409426; doi:10.1016/j.stemcr.2019.01.007)
Supplement: Document S2. Article plus Supplemental Information [file mmc2.pdf]

Stem Cell Reports
Article
OPEN ACCESS

Combined Notch and PDGF Signaling Enhances Migration and Expression of Stem Cell Markers while Inducing Perivascular Cell Features in Muscle Satellite Cells

Mattia Francesco Maria Gerli,[1,2,15] Louise Anne Moyle,[1,9,15] Sara Benedetti,[1,3,4] Giulia Ferrari,[1] Ekin Ucuncu,[1,10] Martina Ragazzi,[1,11] Chrystalla Constantinou,[1,12] Irene Louca,[1,13] Hiroshi Sakai,[5,6,14] Pierpaolo Ala,[7] Paolo De Coppi,[2] Shahragim Tajbakhsh,[5,6] Giulio Cossu,[8] and Francesco Saverio Tedesco[1,7,*]
[1]Department of Cell and Developmental Biology, University College London, WC1E 6DE London, UK
[2]Stem Cell and Regenerative Medicine Section, Great Ormond Street Institute of Child Health, University College London, WC1N 1EH London, UK
[3]Molecular and Cellular Immunology Section, Great Ormond Street Institute of Child Health, University College London, WC1N 1EH London, UK
[4]NIHR Great Ormond Street Hospital Biomedical Research Centre, WC1N 1EH London, UK
[5]Department of Developmental & Stem Cell Biology, Institut Pasteur, 75015 Paris, France
[6]CNRS UMR 3738, Institut Pasteur, 75015 Paris, France
[7]The Dubowitz Neuromuscular Centre, Great Ormond Street Institute of Child Health, University College London, WC1N 1EH London, UK
[8]Division of Cell Matrix Biology and Regenerative Medicine, University of Manchester, M13 9PL Manchester, UK
[9]Present address: Institute of Biomaterials and Biomedical Engineering, University of Toronto, M5S 3E1 Toronto, Canada
[10]Present address: Institut Imagine, INSERM UMR 1163, Paris Descartes-Sorbonne Paris Cité Université, Paris 75015, France
[11]Present address: MolMed S.p.A., 20132 Milan, Italy
[12]Present address: National Heart and Lung Institute, Imperial College London, SW3 6LY London, UK
[13]Present address: Division of Neuroscience and Experimental Psychology, University of Manchester, M13 9PL Manchester, UK
[14]Present address: Division of Integrative Pathophysiology, Proteo-Science Center, Ehime University, Toon, Ehime 791-0295, Japan
[15]Co-first author
*Correspondence: f.s.tedesco@ucl.ac.uk
https://doi.org/10.1016/j.stemcr.2019.01.007

SUMMARY

Satellite cells are responsible for skeletal muscle regeneration. Upon activation, they proliferate as transient amplifying myoblasts, most of which fuse into regenerating myofibers. Despite their remarkable differentiation potential, these cells have limited migration capacity, which curtails clinical use for widespread forms of muscular dystrophy. Conversely, skeletal muscle perivascular cells have less myogenic potential but better migration capacity than satellite cells. Here we show that modulation of Notch and PDGF pathways, involved in developmental specification of pericytes, induces perivascular cell features in adult mouse and human satellite cell-derived myoblasts. DLL4 and PDGF-BB-treated cells express markers of perivascular cells and associate with endothelial networks while also upregulating markers of satellite cell self-renewal. Moreover, treated cells acquire trans-endothelial migration ability while remaining capable of engrafting skeletal muscle upon intramuscular transplantation. These results extend our understanding of muscle stem cell fate plasticity and provide a druggable pathway with clinical relevance for muscle cell therapy.

INTRODUCTION

Skeletal muscle homeostasis and regeneration rely on resident stem cells named satellite cells (SCs), which reside underneath the basal lamina of the myofibers and express the transcription factor *Pax7*. Upon injury, activated SCs generate transient amplifying precursors called myoblasts, which fuse to form multinucleated myofibers (Sambasivan and Tajbakhsh, 2015). The regenerative capacity of SCs has led to the development of cellular therapies to replace lost or damaged muscle (Tedesco et al., 2010). Despite some promising pre-clinical results and a good safety profile, clinical trials based upon intramuscular myoblast transplantation in patients with Duchenne muscular dystrophy (DMD) have reported limited efficacy (Briggs and Morgan, 2013). This outcome has been ascribed to the poor survival of myoblasts, their limited ability to migrate, and the host immune reaction (Partridge, 2002), although this is still a matter of active debate (Skuk and Tremblay, 2014). More recently, local delivery of myoblasts to affected muscles in oculopharyngeal muscular dystrophy patients has shown encouraging results (Perie et al., 2014). Nevertheless, myoblasts are considered unsuitable for systemic delivery, preventing their use for the treatment of patients affected by severe myopathies with widespread muscle involvement such as DMD.

Perivascular cells (pericytes in particular) support skeletal muscle perfusion, development, and regeneration (Birbrair and Delbono, 2015; Cappellari and Cossu, 2013; Murray et al., 2017). Despite evidence in transgenic mice showing that SCs (Relaix and Zammit, 2012) and not pericytes (Guimaraes-Camboa et al., 2017) are required for muscle regeneration, there are reports indicating that pericyte-derived cells can also contribute to skeletal myogenesis, including SC generation and maintenance (Dellavalle et al., 2011, 2007; Kostallari et al., 2015; Sacchetti et al., 2016; Tedesco et al., 2011). Discrepancies among reports may be due to the use of different markers to identify interstitial/perivascular cells

This is an open access article under the CC BY license (http://creativecommons.org/licenses/by/4.0/).

(Tedesco et al., 2017). Importantly, intra-arterial delivery of progenitors derived from skeletal muscle perivascular cells (mainly mesoangioblasts, deriving from *in vitro* expansion of a subset of muscle pericytes) resulted in the colonization of skeletal muscle tissue downstream of the injection site and subsequent amelioration of different animal models of muscular dystrophy (Benedetti et al., 2013). Moreover, a recent first-in-human phase I/IIa clinical trial based on intra-arterial delivery of human leukocyte antigen-matched mesoangioblasts in DMD children has established the safety and feasibility of this procedure (Cossu et al., 2015). While they may be an important source for transplantation, the skeletal myogenic and self-renewing potential of perivascular cells is suboptimal compared with SCs, and their preliminary clinical investigation indicates that further optimization will be needed for muscle cell therapy (Cossu et al., 2015). Therefore, a muscle stem cell harboring SC myogenic and self-renewing capacity combined with the migration ability of perivascular cells could be ideal for muscle cell therapies.

Several groups have shown that the Notch signaling pathway, a key regulator of myogenesis and pericyte function, can alter the behavior of myogenic precursors (Mourikis and Tajbakhsh, 2014; Sainson and Harris, 2008). The Notch ligand delta ligand 1 (DLL1) promotes SC quiescence (Baghdadi et al., 2018) and increases engraftment of canine muscle cells (Parker et al., 2012), whereas DLL4 regulates mouse SC self-renewal (Low et al., 2018; Verma et al., 2018); however, DLL1 and DLL4 alone did not significantly improve engraftment of mouse and human SCs (Sakai et al., 2017). Conversely, Notch depletion leads to SC exhaustion, impairment of muscle regeneration, and reduced engraftment of mesoangioblasts (Bjornson et al., 2012; Mourikis et al., 2012; Quattrocelli et al., 2014; Schuster-Gossler et al., 2007; Vasyutina et al., 2007).

Platelet-derived growth factor (PDGF) signaling also has important roles in regulating smooth and skeletal muscle cell fate. The PDGF signaling pathway comprises the two receptors α (PDGFR-A) and β (PDGFR-B), which bind to ligands PDGF-A/-B/-C/-D as homo- or hetero-dimers (Lu and Li, 2017). PDGF-B is expressed in both SC and pericytes (Pinol-Jurado et al., 2017), affecting their proliferation, migration, recruitment, and fate (Lindahl et al., 1997; Pallafacchina et al., 2010; Sugg et al., 2017; Yablonka-Reuveni et al., 1990). In addition, PDGF-BB is upregulated in dystrophic myofibers and attracts myoblasts (Pinol-Jurado et al., 2017); with a similar mechanism, endothelial cells recruit mural cells via PDGF-BB (Betsholtz, 2004). Importantly, Notch induces PDGFR-B, and this combined signaling directs vascular smooth muscle cell fate choice (Jin et al., 2008).

Previously we reported that mouse embryonic myoblasts undergo a fate switch toward the perivascular lineage following stimulation with DLL4 and PDGF-BB (Cappellari et al., 2013). Although this prior study suggests bidirectional fate plasticity between SCs and pericytes, there is currently no evidence indicating that a similar phenomenon is conserved in adult myogenic progenitors. Here, we provide evidence that adult skeletal muscle SCs gain pericyte properties in response to DLL4 and PDGF-BB treatment, while also re-acquiring a stemness signature.

RESULTS

DLL4 and PDGF-BB Treatment Induces Reversible Changes in Morphology, Proliferation, and Differentiation of Adult Murine Satellite Cell-Derived Myoblasts

To determine whether adult SCs respond to the activation of Notch and PDGF pathways, primary SC-derived myoblast cultures (hereafter referred to as SCs) were established from wild-type mice (Figure S1A) and cultured on collagen-coated dishes (to aid attachment) or seeded on DLL4-coated dishes supplemented daily with PDGF-BB. After 1 week of treatment, the morphology of the treated SCs was compared with untreated control SCs, revealing a change from a round to a more elongated morphology (Figures 1A and 1B).

Notch activity controls SC activation, proliferation, and quiescence (Bjornson et al., 2012; Brohl et al., 2012; Mourikis et al., 2012). Consistent with these reports, we observed reduced proliferation of SCs exposed to the treatment (Figure 1C). This effect was reverted by discontinuation of the treatment. Interestingly, reduced cell proliferation was also observed when the treatment was commenced on cells that were previously expanded for 2 weeks in control conditions, indicating that the responsiveness to treatment was not impaired with extensive culture (Figures 1D–1F). Specifically, proliferation was analyzed by functional 5-ethynyl-2′-deoxyuridine (EdU) incorporation assay and nuclear Ki67 immunofluorescence staining. Both assays showed a decrease in proliferation from control to treated SCs: 28.19% vs 10.94% EdU incorporation ($p = 0.021$, $N = 3$) and 45.60% vs 20.44% Ki67 positivity ($p = 0.047$, $N = 3$).

Notch activation inhibits myogenesis *in vitro* both in embryonic myoblasts and adult SCs (Conboy and Rando, 2002; Kopan et al., 1994; Mourikis and Tajbakhsh, 2014). To investigate if this mechanism is triggered upon treatment with DLL4 and PDGF-BB, cells were differentiated into skeletal myotubes and immunoassayed for myosin heavy chain (MyHC) (Figures 1G and 1H). A significant reduction in myogenic differentiation upon treatment was observed, from 71.5% to 37.9% ($p = 0.031$, $N = 3$ mice in 4 experiments), reflecting the role of Notch signaling in inhibiting myogenesis. This differentiation

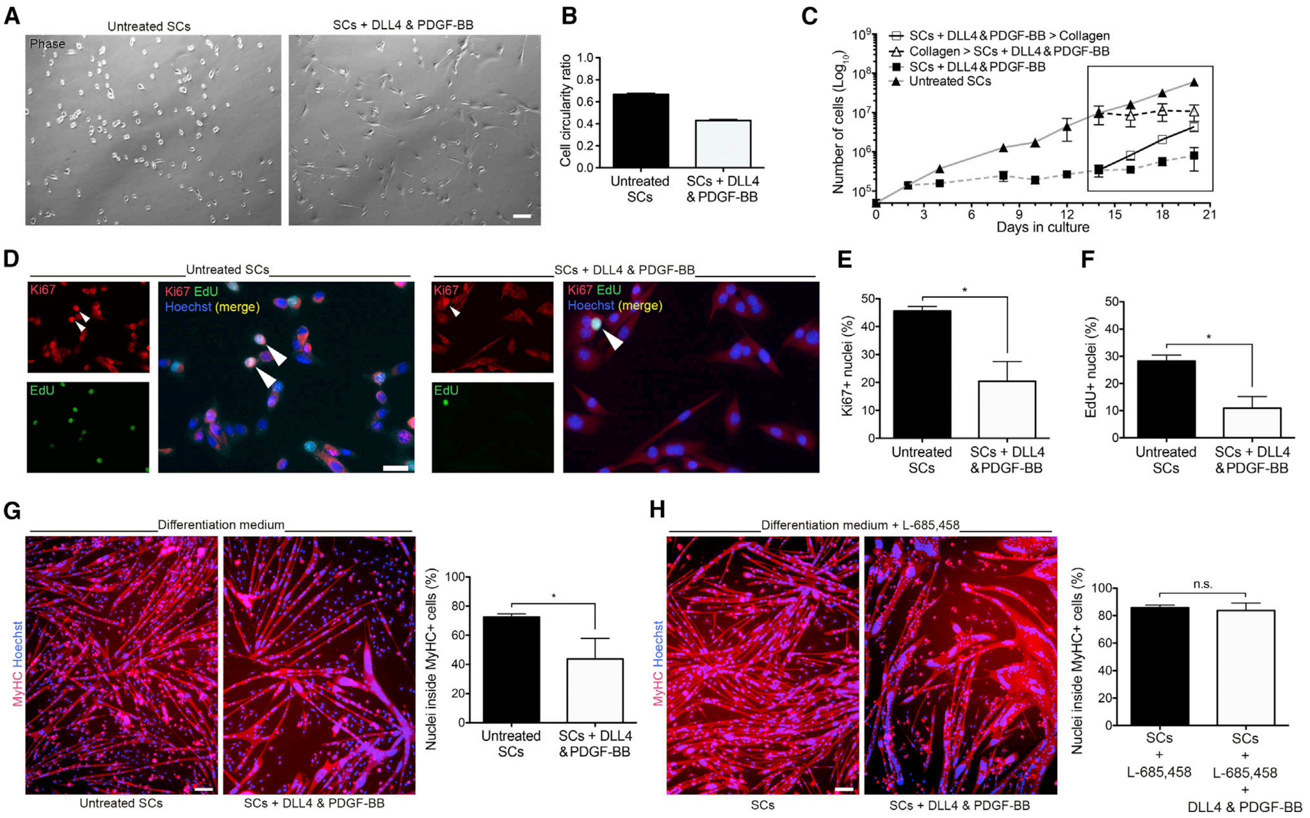

Figure 1. Morphology, Proliferation, and Differentiation of DLL4 and PDGF-BB-Treated SCs

(A) Phase contrast images of untreated and DLL4 and PDGF-BB-treated SCs isolated from CD1 mice.

(B) Graph quantifies circularity ratio, where 1 = circle and 0 = line (n = 3).

(C) Proliferation curves of untreated and treated SCs over time (n = 3). Box highlights treatment switch.

(D–F) Immunofluorescence analysis of SCs isolated from *TN-AP^{cre}:TdTomato* mice expanded for 2 weeks prior to treatment, or maintained in untreated conditions. Cells pulsed for 2 h with EdU and co-immunostained with Ki67 (arrowheads: nuclear signal) (N = 3) (D). Quantified in (E and F).

(G) Immunofluorescence images of untreated and treated SCs differentiated into myotubes in low mitogen medium for 4 days and immunostained for myosin heavy chain (MyHC) and Hoechst (N = 3 mice and 4 experiments).

(H) Untreated and treated SCs differentiated in low mitogen medium supplemented with 660 ng/mL of the γ-secretase inhibitor L-685,458 to inhibit Notch signaling (N = 3).

Data: means ± SEM. Statistical significance based on paired (E and F) or unpaired (G and H) Student's t test; *$p < 0.05$; n.s., not significant. Scale bars, 25 μm (D) and 100 μm (A, G, and H).

impairment was reverted by blocking the Notch cascade with the γ-secretase inhibitor L685,458 (N = 3), confirming the Notch dependency of this phenomenon (Figures 1G and 1H).

DLL4 and PDGF-BB Treatment Induces Transcription of Perivascular and SC Genes

To understand the mechanism behind the observed proliferation and differentiation changes, a series of real-time qPCR analyses was performed to investigate the variation in the gene expression profile of SCs upon DLL4 and PDGF-BB treatment. Preliminary experiments performed with SCs isolated with the pre-plating methodology from CD1 mice (Figure S1A) suggested that a number of transcripts associated with Notch and PDGF pathways, as well as myogenic and smooth muscle programs, responded to treatment (Figure S1B).

To ensure that the observed gene expression profile was directly elicited by DLL4 and PDGF-BB on bona fide SCs and not on a possible minority of contaminating cells, we used SCs isolated from *Tg:Pax7-nGFP* mice (Sambasivan et al., 2009) (Figure 2A). Analysis of the expression of *Notch1*, the main receptor for DLL4, and of *Pdgfrb*, showed significant transcriptional upregulation, confirming that treatment with DLL4 and PDGF-BB activated the expected signaling pathways (p = 0.038 and p = 0.017, respectively,

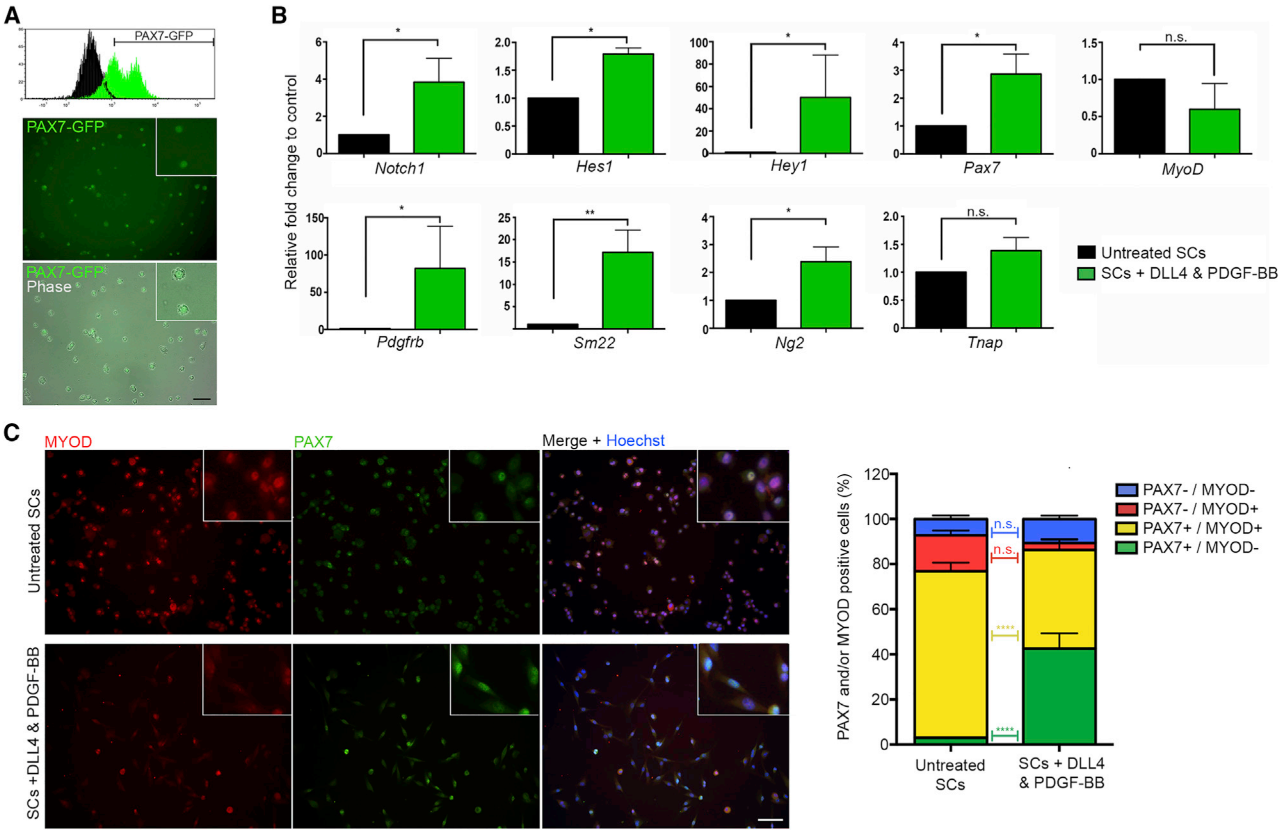

Figure 2. Gene Expression Analyses of Treated SCs via Real-Time qPCR and Immunofluorescence

(A) Histogram view of the FACS purification of PAX7-GFP+ SCs and resulting pure population in phase contrast and GFP images.

(B) Real-time qPCR analysis of untreated (black bars) and DLL4 and PDGF-BB-treated (green bars) PAX7-GFP+ SCs. Graphs show fold change to control conditions, statistical significance based on ΔCt (N = 4).

(C) Left panel: PAX7-GFP+ SCs cultured in control (untreated) or DLL4 and PDGF-BB conditions and immunostained with MYOD (red) and PAX7 (green) antibodies. Right graph quantifying the relative percentages of PAX7/MYOD +/− cells in each condition (N = 3).

Data: means ± SEM. Statistical significance based on paired Student's t tests (B) or two-way ANOVA with Bonferroni's multiple comparison (C). *$p \leq 0.05$, **$p \leq 0.01$, ****$p \leq 0.0001$, n.s., not significant. Scale bars, 50 μm (A and C). See also Figure S1.

Figure 2B). This was also confirmed by increased expression of Notch targets *Hes1* (p = 0.028) and *Hey1* (p = 0.022). Notch induces *Pdgfrb* expression in vascular smooth muscle cells (Jin et al., 2008), which may explain the remarkable induction of *Pdgfrb*. Interestingly, the treatment induced genes usually more expressed in smooth muscle cells and pericytes, including *Ng2* (p = 0.024) and *Sm22* (p = 0.003), while also increasing expression of the SC marker *Pax7* (p = 0.032). Finally, quantification of alkaline phosphatase (AP) activity, which is associated with myogenic pericytes (Dellavalle et al., 2011), suggested upregulation in treated cells (Figure S1C).

We then measured PAX7 and MYOD proteins in SCs treated with DLL4 and PDGF-BB (Figure 2C, N = 3). PAX7 marks quiescent, self-renewing, and primed SCs, whereas MYOD is associated with committed progenitors (i.e., myoblasts) (Zammit et al., 2004). Similar to what was observed at the mRNA level, the percentage of PAX7+/MYOD– cells increased from 2.97% in untreated cells to 42.47% in treated cells (p < 0.0001). Conversely, the percentage of PAX7+/MYOD+ SCs decreased with DLL4 and PDGF-BB treatment, from 73.90% to 43.79% (p < 0.0001), suggesting that treated cells retained a more "stem-like" state.

For this treatment to be applicable to human cells, response needs to be maintained following expansion in culture. To test this, we assessed whether SCs were responsive to treatment when expanded for 2 weeks prior to treatment, revealing an increase in the proportion of PAX7+/MYOD– SCs (p = 0.001, N = 4; Figure S1D).

Finally, we observed that the morphological changes elicited in treated SCs correlated to an ability of the cells to adhere directly to plastic, rather than requiring collagen coating. To confirm that the effect of DLL4 and PDGF-BB treatment was independent of collagen coating, SCs from

Tg:Pax7-nGFP mice were expanded for 1 week in control and treatment conditions on collagen-coated or uncoated culture dishes: proliferation kinetics and mRNA expression analyses revealed that the effect was independent of coating (Figures S1E and S1F; N = 3). Taken together, these results show that DLL4 and PDGF-BB treatment induces a perivascular-like gene expression profile in SCs while maintaining or inducing a signature associated with their self-renewal.

DLL4 and PDGF-BB-Treated SCs Gain Properties of Perivascular Cells

We next aimed to test whether the expression of perivascular markers on SC treatment with DLL4 and PDGF-BB was also accompanied by functional pericyte properties. Pericytes contribute to the stabilization of endothelial networks in hydrogels on vascular endothelial growth factor (VEGF) stimulation (Cappellari et al., 2013). To determine whether DLL4 and PDGF-BB treatment conferred this functional property to SCs, treated cells were co-cultured with human umbilical vein endothelial cells (HUVECs) in Matrigel and stimulated with VEGF. To allow cell tracing in co-culture, SCs were isolated from *Tg:CAG-EGFP* mice, which ubiquitously express the green fluorescent protein (GFP) (Okabe et al., 1997). After 24 h in culture, endothelial networks showed limited branching and partial disaggregation when cultured alone or with untreated SCs; however, networks showed increased branching and stability when co-cultured with SCs exposed to DLL4 and PDGF-BB, similarly to control skeletal muscle pericytes (Figures 3A–3D).

A characteristic of pericyte-derived mesoangioblasts is their ability to migrate outside blood vessels upon intravascular delivery in models of muscular dystrophy (e.g., Giannotta et al., 2014; Tedesco et al., 2011). To investigate whether DLL4 and PDGF-BB-treated SCs acquired this property, we first performed a transwell assay on an H5V murine endothelial cell monolayer and measured the number of control or treated SCs that migrated through the endothelial layer after 6 h. Notably, treated cells had about a 3-fold increase in migration ability compared with untreated SCs ($p < 0.01$, N = 3; Figure 3E). Subcutaneously injected DLL4 and PDGF-BB-treated SCs transplanted into Matrigel plugs also appeared to migrate more toward CD31/PECAM+ host blood vessels, suggesting a response to chemotactic signals from the native endothelium (Figure S2, N = 2).

Proof-of-principle *in vivo* testing was then performed to determine whether improved migration through an endothelial monolayer *in vitro* resulted in SCs with the capacity to cross an actual blood vessel wall. To easily visualize cells, they were transduced with a lentivirus carrying a nuclear LacZ cDNA (*nLacZ*), which enables β-galactosidase (β-GAL)-mediated identification of donor cells with the X-gal reaction. A total of 10^6 control or treated *GFP+/nLacZ+* SCs were injected into the femoral arteries of dystrophic immunodeficient α-sarcoglycan-null/scid/beige mice (*Sgca^{tm1Kcam}Prkdc^{scid}Lyst^{bg}*, *Sgca-null/scid/beige* hereafter; Tedesco et al., 2012) as reported previously (Gerli et al., 2014). This strain was chosen as it displays a more severe dystrophic phenotype compared with the *mdx* mouse and does not have revertant fibers. Prior to transplantation cells were stained with X-gal to determine the efficiency of transduction (84% X-gal+). Three weeks after transplantation, scattered GFP+/X-gal+ donor-derived nuclei were observed in muscles downstream of the injection site in limbs transplanted with DLL4 and PDGF-BB-treated SCs, with muscles receiving untreated SCs showing little to no signal (Figure 4A). The relatively low frequency of the event made it difficult to quantify with conventional methods (e.g., nuclei/section); however, the average X-gal+ area in the medial aspect of the hindlimb muscles increased from 0.02 mm^2 to 0.1 mm^2 ($p = 0.028$, N = 4, Figure 4A), suggesting an acquired ability of treated SCs to cross the blood vessel wall upon intra-arterial delivery. X-gal staining of filter organs (i.e., liver, spleen, kidneys, and lungs) showed no donor nuclei in mice receiving control or DLL4 and PDGF-BB-treated SCs intra-arterially (data not shown). These experiments show that DLL4 and PDGF-BB treatment induces perivascular properties to SCs such as endothelial branch stabilization, as well as improved migration *in vitro* and *in vivo*.

DLL4 and PDGF-BB-Treated Mouse SCs Engraft Skeletal Muscle following Intramuscular Transplantation

To investigate if the increased migration resulted in improved *in vivo* engraftment while retaining their myogenic memory, 1×10^6 GFP+/β-GAL+ SCs were transplanted intramuscularly into the tibialis anterior (TA) muscle of *Sgca-null/scid/beige* mice (N = 5, Figures 4B–4F). Three weeks after transplantation, explanted muscles were cryosectioned and co-stained for X-gal and α-sarcoglycan (SGCA) to detect donor-derived cells and their contribution to host tissues. This analysis revealed that the number of engrafted cells significantly increased with DLL4 and PDGF-BB treatment ($p = 0.047$, Figure 4B). Although TA muscles engrafted with treated SCs appeared to have more cells per section and a greater migration throughout the muscle than control SCs (Figure 4C), the percentage of X-gal+ nuclei located within myofibers (i.e., donor myonuclei) was unchanged with the treatment (Figure 4D). Finally, a similar number of SGCA+ myofibers was also observed (Figures 4E and 4F), indicating full differentiation capacity of treated SCs. In parallel, tumor formation assays based on subcutaneous injection of 2×10^6 treated SCs were performed to assess safety of the signaling manipulation. After

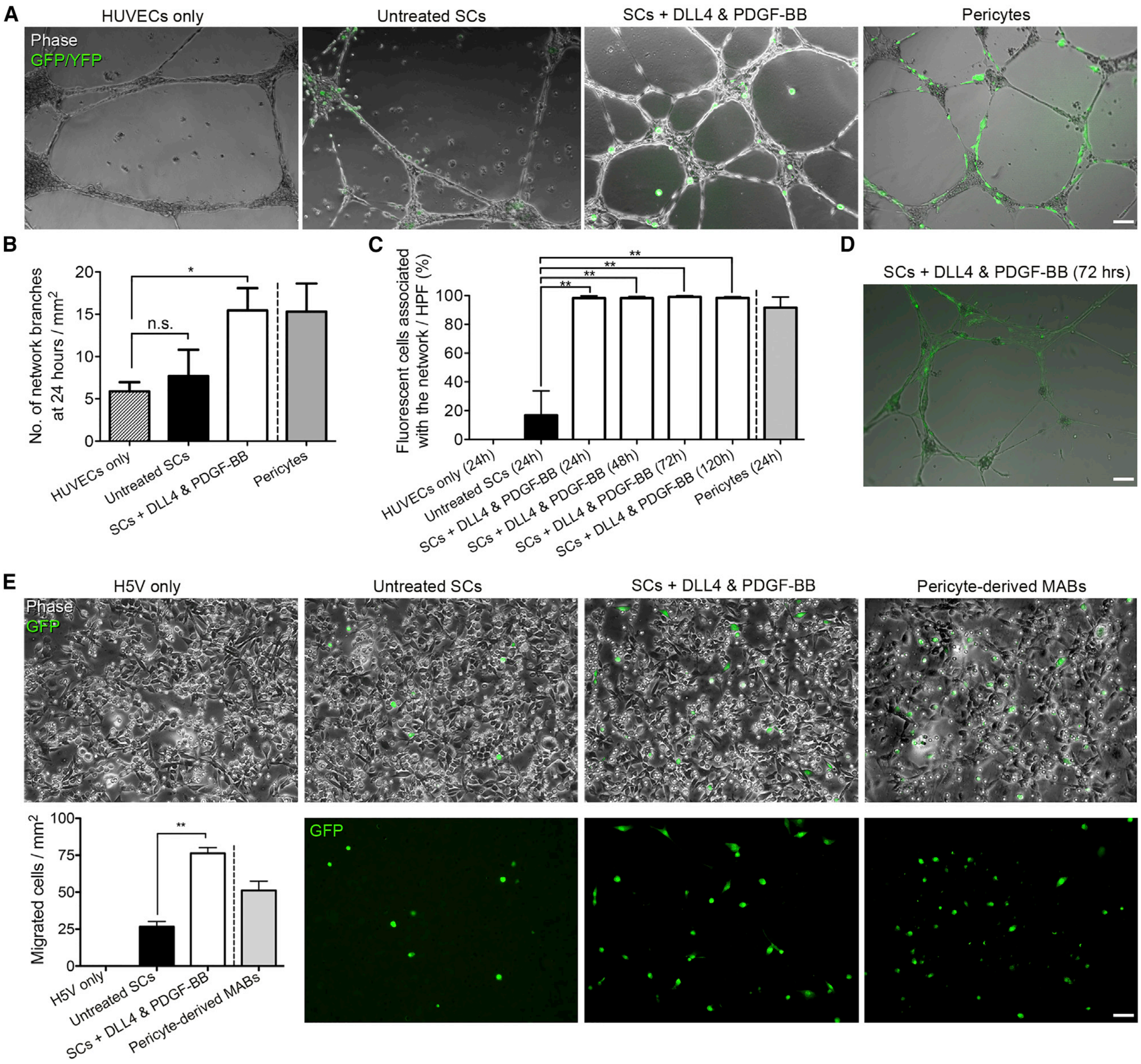

Figure 3. *In Vitro* Assessment of Endothelial Network Association and Migration Properties of DLL4 and PDGF-BB-Treated SCs

(A) Representative images from endothelial network formation assays at 24 h with GFP+ untreated or DLL4 and PDGF-BB-treated SCs and control yellow fluorescent protein (YFP)+ primary muscle pericytes (technical control, from *Tg:TN-AP-CreERT2*:*R26R-EYFP* mice) stimulated with VEGF in the presence of HUVECs.

(B) Graph depicts quantification of network branches per mm^2 at 24 h in the same experimental groups shown in (A) (N = 4).

(C and D) Graph quantifies GFP+ SCs and YFP+ pericytes associated with the network per high-power field (HPF = 1.5 mm^2) over time (N = 4); representative image of treated cells at 72 h shown in (D).

(E) Images of the lower side of transwell membranes from GFP+ untreated or treated SCs and control pericyte-derived mesoangioblasts (MABs) seeded on a monolayer of H5V murine endothelial cells. Graph quantifies the average number of cells/mm^2 that migrated through the endothelial layer after 6 h (N = 3).

Means ± SEM; statistical significance based on one-way ANOVA with Dunnett's multiple comparison test (B and C) or paired t test (E); *$p < 0.05$, **$p \leq 0.01$. Scale bars, 100 μm.

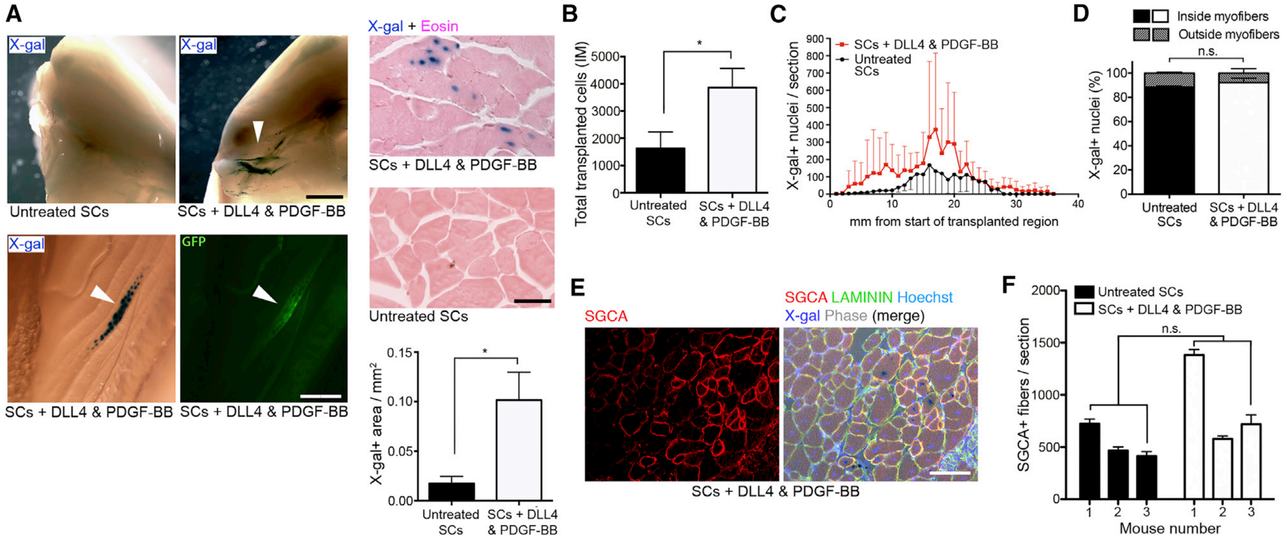

Figure 4. *In Vivo* Assessment of Migration and Engraftment of DLL4 and PDGF-BB-Treated SCs

(A) *In vivo* analysis of limb muscles from *Sgca-null/scid/beige* mice intra-arterially transplanted with 10^6 GFP+/β-GAL+ control or DLL4 and PDGF-BB-treated SCs after 3 weeks. Upper panel: exposure to X-gal revealed engraftment of β-GAL+ donor cells (blue; arrowhead) in the knee region and lower limb muscles of mice transplanted with DLL4 and PDGF-BB-treated SCs. Lower panel: assessment of X-gal in the gastrocnemius muscle of mice transplanted with treated SCs, revealing nuclear localization of β-GAL in a GFP+ myofiber (arrowheads). Graph quantifies the area of X-gal+ signal in muscles intra-arterially transplanted with treated or untreated SCs (lateral view of knee region) normalized to the volume of cell suspension delivered (N = 4).

(B) Quantification of transplanted cells that engrafted intramuscularly injected TA muscles (N = 5).

(C) Distribution across muscle length of X-gal+ nuclei per section of TA muscles transplanted with treated and untreated SCs (N = 5).

(D) Quantification of the localization (i.e., inside or outside myofibers) of X-gal+, treated/untreated, donor-derived nuclei in transplanted muscles (N = 5).

(E) Immunofluorescence panel showing a representative *Scga-null/scid/beige* muscle transplanted with 10^6 GFP+/*nLacZ*+ DLL4 and PDGF-BB-treated SCs intramuscularly 3 weeks before explant. SGCA (red) and X-gal (violet): donor-derived fibers and nuclei, respectively. LAMININ (green) and Hoechst (blue): extracellular matrix and nuclei, respectively.

(F) Quantification of the experiment in (E) showing the average number of donor-derived fibers from three mice.

Data: means ± SEM. Statistical significance based on unpaired Welch's t test. $^*p < 0.05$; n.s., not significant. Scale bars, 2 mm (A, left panel), 250 μm (A, middle panel), 50 μm (A, right panel), and 50 μm (E).

a minimum 2-month follow-up, no tumors were seen (N = 5 mice), indicating that the treatment did not result in oncogenic transformation of treated cells. These data show that, upon treatment with DLL4 and PDGF-BB, mouse SC-derived myoblasts have improved engraftment in host skeletal muscle than untreated cells, although with comparable contribution to myofiber repair/regeneration.

Human SC-Derived Myoblasts Respond to DLL4 and PDGF-BB Stimulation

Translation of this strategy into future pre-clinical studies requires evidence that the mechanism is conserved also in human SC-derived myoblasts (hereafter referred to as HuSCs). To this aim, primary human muscle biopsy-derived SCs from five individuals (HuSCs 1–5) were isolated and purified by fluorescence-activated cell sorting (FACS) for CD56 positivity, which marks HuSCs (Boldrin et al., 2010). Purified SCs were expanded and treated with human DLL4 and PDGF-BB for 7 days or expanded in control conditions. Proliferation analyses (Figures 5A–5C) revealed that, unlike murine SCs, the percentage of EdU+ cells was unaffected by DLL4 and PDGF-BB treatment (Figure 5B). The exception to this was donor no. 4, where a significant increase was observed ($p = 0.002$). The percentage of Ki67+ cells was assessed in three HuSC preparations to confirm EdU analyses, which, although variable between preparations, was unaffected when results were averaged (Figure 5C).

Analysis of transcriptional changes elicited by DLL4 and PDGF-BB treatment showed activation of Notch signaling via increased *HEY1* (HuSC3 $p = 0.006$, HuSC5 $p = 0.028$) and *HES1* (HuSC3 $p > 0.0001$, HUSC5 $p = 0.003$), although differences in expression levels were observed between the preparations assessed (Figure 5D). A trend in the increase of *PDGFRB* transcripts was also observed, although not statistically significant. As with murine SCs, myogenic markers

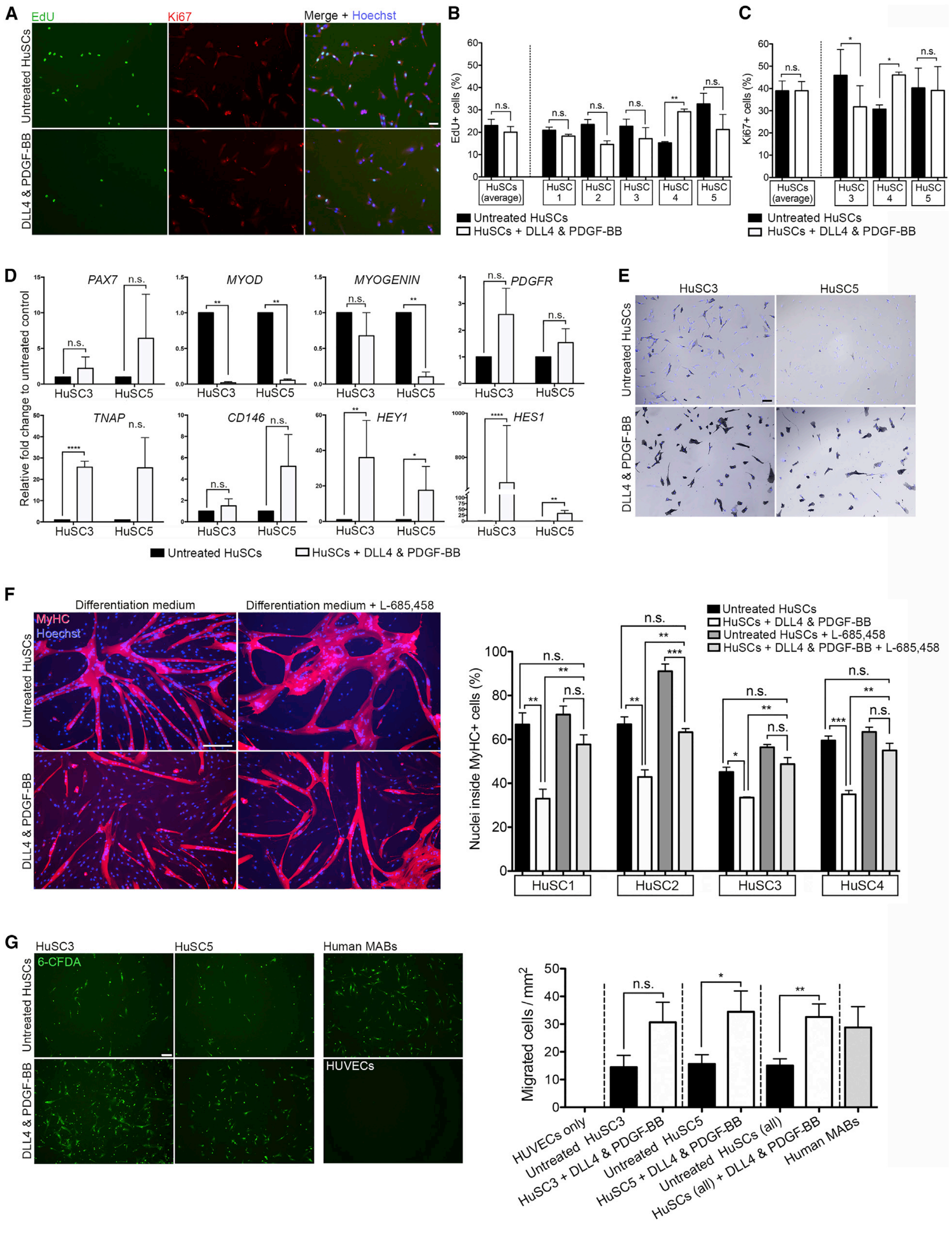

(legend on next page)

MYOD (HuSC3 p = 0.006, HuSC5 p = 0.008) and *MYOGENIN* (HuSC5 p = 0.007) were decreased, whereas the pericyte marker *TNAP* was increased (p < 0.0001). This correlated with an increase in AP enzymatic activity (Figure 5E). *PAX7* expression did not increase significantly with treatment, although all samples expressed very low baseline levels, likely due to cell expansion in culture, or distinct regulation of this gene in the human compared with mouse (Figure 5D).

We then tested HuSC differentiation in response to DLL4 and PDGF-BB treatment. To determine whether any changes were due to Notch, cells were differentiated in low mitogen medium for 4 days in the presence or absence of γ-secretase inhibitor (Figure 5F). In all preparations assessed, DLL4 and PDGF-BB treatment reduced myogenic differentiation, which was rescued by inhibiting Notch signaling (Figure 5F).

To assess whether transcriptional changes induced by DLL4 and PDGF-BB treatment had functional consequences in HuSCs, transmigration ability was assessed via transwell assay on a monolayer of HUVECs (Figure 5G, N = 3). After 8 h, treated cells showed an average 2-fold overall increase in migration capacity, from 15.02 to 32.54 cells/mm^2 (p = 0.008), although some variability was observed among the lines assessed (e.g., p = 0.144 for HuSC3 and p = 0.046 for HuSC5). Taken together, these results indicate that DLL4 and PDGF-BB induce cell fate changes and improve migration also in HuSCs, laying the foundation for further studies on this strategy for human muscle cell therapy.

DISCUSSION

In this work, we hypothesized that cell fate determinants involved in smooth and skeletal muscle lineage determination in the embryo may exert a similar effect also on adult SCs, and that their modulation could be exploited to improve skeletal muscle cell therapies. Direct lineage reprogramming (also known as trans-differentiation) is the conversion of one functional cell type to another fate without an intermediate pluripotent step (Xu et al., 2015). Here, we show that a phenomenon resembling reversible lineage reprogramming takes place in adult SCs in response to DLL4 and PDGF-BB stimulation. Adult SC-derived myoblasts treated with DLL4 and PDGF-BB increase expression of perivascular genes. At variance with their embryonic counterpart (Cappellari et al., 2013), adult SCs acquired also an expression profile suggestive of self-renewing SCs (i.e., high PAX7 and low MYOD). However, the co-expression of SC and perivascular markers indicates that the treatment does not trigger a complete trans-differentiation, but rather a stimulus-dependent reprogramming into a hybrid cell type. Our isolation procedures (FACS purification using PAX7-GFP for murine cells and CD56 for their human counterpart) minimize the possibility that the observations are due to contaminating cells.

Similar to bona fide pericytes, treated SCs become associated with endothelial networks (Armulik et al., 2005). Notably, transwell assays and *in vivo* experiments showed improved ability of treated SCs to cross endothelial barriers, suggesting acquisition of a similar machinery that pericyte-derived mesoangioblasts might use to cross the blood vessel wall (Giannotta et al., 2014).

Manipulating Notch signaling affects SC differentiation and transplantation ability. Recently, it was shown that while Jagged1, DLL1, or DLL4 alone can maintain a stem-like phenotype in SCs grown *ex vivo*, this does not correlate with an increased efficiency to generate myofibers upon transplantation (Sakai et al., 2017). Interestingly, our

Figure 5. Effect of DLL4 and PDGF-BB Treatment on Human SC-Derived Myoblasts

(A) Representative images of CD56+ FACS-purified human SC-derived myoblasts from two individuals (HuSC 3 and 5) expanded in control conditions (upper panel) or treated with DLL4 and PDGF-BB (lower panel) for 1 week, then pulsed for 2 h with EdU (green) and co-immunostained with Ki67 (red).

(B) Quantification of the percentage of EdU+ cells (N = 5).

(C) Quantification of Ki67+ cells (N = 3).

(D) Real-time qPCR analysis of myogenic (*PAX7*, *MYOD*, and *MYOGENIN*), perivascular (*PDGFRB*, *TNAP*, and *CD146*) and Notch signaling (*HES1* and *HEY1*) genes in response to DLL4 and PDGF-BB treatment in HuSC 3 and 5 (n = 3 per line). Graphs show fold change to control conditions, statistical significance based on ΔCt.

(E) Phase contrast images of AP enzymatic activity (violet) and Hoechst (blue) in untreated and treated HuSCs.

(F) Differentiation of HuSCs in response to DLL4 and PDGF-BB treatment and Notch inhibition. Cells expanded in control or treatment conditions for 1 week and induced to differentiate for 4 days in the presence or absence of L-685,458. Pictures representatives from HuSC1, and graphs quantify the percentage of MyHC+ cells (N = 4).

(G) Representative images of the lower chambers of transwell assays. Untreated/treated HuSCs and human muscle pericyte-derived mesoangioblasts (MABs; technical control) were seeded for 8 h on HUVECs. Right graph: number of 6-CFDA+ migrated cell/mm^2 in each condition. N = 3; means ± SEM.

Statistical significance based on paired Student's t test (B, C and cell-line-specific data in G), unpaired Student's t test (D and pooled data in G) or one-way ANOVA with Tukey's multiple comparison (F). *p < 0.05, **p $\leq$ 0.01, ***p $\leq$ 0.001, ****p $\leq$ 0.0001; n.s., not significant. Scale bars, 50 μm (A), 75 μm (F), and 100 μm (E and G).

results indicate that, when combined with PDGF-BB, DLL4 induces perivascular features in SCs and increases their engraftment into host skeletal muscle, albeit with a similar contribution to myofibers. We hypothesize that this might be due to increased migration throughout the host muscle resulting in fusion of more donor cells with the same myofibers. Hence, this migration-enhancing strategy preserves the overall SC engraftment and differentiation potential without altering their safety profile, as shown by lack of oncogenic transformation in transplantation experiments and tumorigenesis assays.

It is thought-provoking to speculate that satellite and perivascular cells might retain bidirectional plasticity in adulthood due to a residual developmental memory from a common precursor. It has been shown that skeletal and vascular smooth muscle cells have a common PAX3+ progenitor in the dermomyotome (Esner et al., 2006; Goupille et al., 2011; Lagha et al., 2009). At later stages of development, it has been shown that embryonic myoblasts and skeletal muscle pericytes maintain the ability to transdifferentiate toward a smooth or skeletal muscle fate, in response to DLL4, PDGF, and Noggin signaling, respectively (Cappellari and Cossu, 2013). Although experiments in mice challenge the model of endogenous pericytes as tissue-resident progenitors (Guimaraes-Camboa et al., 2017), there is evidence that human skeletal myogenic progenitors are associated with blood vessel walls *in vivo* in perivascular locations typical of mural cells/pericytes or adventitial cells of muscular veins (Dellavalle et al., 2007; Sacchetti et al., 2016). This population has been hypothesized to represent either a subset of myogenic progenitors recruited to separate niches based on proximity (i.e., satellite vs vascular niche), or, alternatively, to be a class of progenitors upstream of SCs. Both models would fit with our data, with DLL4 and PDGF-BB treatment either mimicking *in vitro* what hypothesized by the first model or bringing the cells back to their primitive nature, as per the second model. Although *ex vivo* culture is a key procedure used in this study, previous *in vivo* evidence in the developing embryo underpins this skeletal muscle-vascular crosstalk in adult SCs. Specifically, we demonstrated that SCs are responsive to DLL4 and PDGF-BB treatment irrespective of mouse strain (CD57BL/6 and CD1), isolation procedure (pre-plating or PAX7-GFP sorting), tissue culture substrate, timing of treatment (i.e., immediately upon isolation or following expansion) or species (mouse and human).

Future applications of this strategy include its extension to pluripotent cell-derived myogenic cells, which we and others are developing as a promising tool for regenerative medicine (Loperfido et al., 2015). Taken together, our data indicate that SC plasticity toward the perivascular lineage is conserved in adulthood and opens the possibility to exploit this phenomenon to generate clinically relevant stem cell populations for future cell therapies of muscle disorders.

EXPERIMENTAL PROCEDURES

Animals

Mouse strains used in this study: *Tg:Pax7-nGFP* F1:C57BL/6:DBA2 expressing nuclear-localized EGFP in *Pax7*-expressing cells (Sambasivan et al., 2009); *Tg:TN-AP-CreERT2* (Dellavalle et al., 2011); Gt(ROSA)26Sor$^{tm14(CAG\text{-}tdTomato)Hze}$, a *Td:Tomato* reporter in *Cre*-expressing cells (Madisen et al., 2010) hereafter referred to as *Td:Tomato*; *R26*R-*EYFP*, a reporter expressing cytoplasmic YFP in *Cre*-expressing cells (Srinivas et al., 2001); *Tg:CAG-EGFP* mice expressing ubiquitous GFP (Okabe et al., 1997); CD1 and *Sgca-null/scid/beige* mice (*Sgca*^{tm1Kcam}*Prkdc*^{scid}*Lyst*^{bg}; Tedesco et al., 2012). Housing of all mice (except *Tg:Pax7-nGFP*) and all experimental procedures were performed in accordance with British law under the provisions of the Animals (Scientific Procedures) Act 1986, as approved by the University College London Ethical Review Process committees and under UK Home Office Project Licence 70/8566. Surgery was performed under isoflurane anesthesia and every effort made to minimize pain and suffering, including use of analgesia. *Tg:Pax7-nGFP* mice were bred at the Institut Pasteur (Paris, France), in compliance with European legislation and under the approval of the institutional ethical committee.

Cell Isolation, Culture and Assays

Primary murine SCs and pericytes were isolated from dissected adult skeletal muscles and cultured as detailed in Supplemental Experimental Procedures. HuSCs were obtained from the MRC Neuromuscular Center Biobank (UCL, London, UK; Research Ethics Committee reference no. 06/Q0406/33). Human cell work was conducted under the approval of the NHS Health Research Authority Research Ethics Committee reference no. 13/LO/1826; IRAS project ID no. 141100. Additional details, including full information on cell culture, purification and protocols used in morphological, proliferation, differentiation, network formation, migration, and transplantation assays are available in Supplemental Experimental Procedures.

DLL4 and PDGF-BB Treatment

Murine recombinant DLL4 (R&D Systems; 1389-D4-050) was resuspended to a concentration 10 μg/mL in sterile PBS containing 1% wt/vol BSA. Flasks were coated with DLL4 at 37°C for 45 min before seeding cells. Cultures were supplemented daily with 50 ng/mL of recombinant PDGF-BB (Sigma; P4056) resuspended in 0.1% BSA/4 mM HCl/PBS. For human myoblast treatment, recombinant human DLL4 (R&D Systems; 1506- D4-050) and human PDGF-BB (R&D Systems; 220-BB-050) were dissolved and treated as murine proteins to final concentrations of 10 μg/mL and 100 ng/mL, respectively. Cells were assessed after at least 7 days of treatment, by which time they had been passaged at least once on fresh DLL4-coated plates.

Immunofluorescence and Enzymatic Staining

Cells and tissue sections were fixed in 4% paraformaldehyde, permeabilized using 0.1% Triton and 1% BSA PBS and blocked in 10% donkey or goat serum in PBS for 60 min. Samples were then incubated overnight at 4°C with the following primary antibodies: mouse anti-MyHC (DSHB MF20, 1:10), mouse anti-CD31/PECAM (DSHB P2B1, 1:2), mouse anti-PAX7 (DSHB, 1:1), rabbit anti-Ki67 (Leica NCL-Ki67p, 1:1,000), mouse anti-α-sarcoglycan (anti-SGCA, Novocastra NCL-a-SARC, 1:150 and Abcam AB49451, 1:150), rabbit anti-SGCA (Abcam EPR14773, 1:1,000 on unfixed sections) rabbit anti-LAMININ (Abcam AB11575, 1:300), rabbit anti-MYOD (Santa Cruz Biotechnology M-318, 1:300). Samples were then incubated with fluorescently-conjugated donkey or goat secondary antibodies (Molecular Probes, Alexa Fluor series, 1:500). Hoechst 33342 (Fluka B2261) was used to counterstain nuclei. AP and X-gal staining were performed by incubating cells at room temperature or 37°C, respectively, until a colorimetric change was observed (Roche NBT/BCIP-11681451001 and Sigma 3117073001).

Gene Expression Analyses

RNA extraction was performed with RNeasy kits (QIAGEN), yield and purity assessed with a Nanodrop spectrophotometer and reverse transcribed into cDNA with the Improm-II Reverse Transcription System kit (Promega; A3800). Relative gene expression values were measured by real-time qPCR using the SYBR Green Real-Time Master Mix (Promega; A600A) on a Bio-Rad CFX96 machine. Real-time qPCRs were performed in triplicate on samples from at least three independent experiments. *Gapdh* and *RPLPO* were used as housekeeping genes for murine and human experiments, respectively. Data presented as means ± SEM of the fold change, normalized to control SCs. Significance was assessed on the delta Ct values using Student's t test assuming two-tailed distribution and equal variances. Sequences of primers available in Supplemental Experimental Procedures.

Statistics

Statistical testing was performed using Microsoft Excel and GraphPad Prism software. N values and experimental replicates are detailed in figure legends. "N" refers to independent experiments or animals (mice or litters); "n" refers to technical replicates. Graphs display means ± SEM unless otherwise stated. Statistical testing was based on t tests or one-way ANOVA with specific *post-hoc* comparison tests as detailed in figure legends.

SUPPLEMENTAL INFORMATION

Supplemental Information includes Supplemental Experimental Procedures and two figures and can be found with this article online at https://doi.org/10.1016/j.stemcr.2019.01.007.

AUTHOR CONTRIBUTIONS

Conceptualization, F.S.T., M.F.M.G., and L.A.M.; Methodology, M.F.M.G., L.A.M., S.B., E.U., M.R., C.C., I.L., H.S., and F.S.T.; Investigation, M.F.M.G., L.A.M., S.B., G.F., and F.S.T.; Writing – Original Draft, M.F.M.G., L.A.M., and F.S.T.; Writing – Review & Editing, M.F.M.G., L.A.M., G.C., S.T., and F.S.T.; Funding Acquisition, F.S.T.; Resources, G.C., S.T., P.A., P.D.C., and F.S.T.; Supervision and Coordination, F.S.T.; M.F.M.G. and L.A.M. contributed equally; S.B., G.F., and E.U. contributed equally.

ACKNOWLEDGMENTS

We are grateful to E. De Marco, E. Tagliafico, E. Tenedini, A. Moreno-Fortuny, J. Lane, A. Aamir, M. Loperfido, G. Maroli, O. Cappellari, F. Pucci, M. Digiovangiulio, A. Eddaoudi, and S. Canning for experimental support and helpful discussions. The Graphical Abstract was created using Servier Medical Art (https://smart.servier.com) in accordance with a Creative Commons Attribution 3.0 Unported License (https://creativecommons.org/licenses/by/3.0/). This work was funded by the European Union's 7th Framework Program for research, technological development and demonstration under grant agreement no. 602423 (PluriMes). Work in the Tedesco laboratory was also funded by the European Research Council (7591108 – HISTOID), Fundació la Marató de TV3 (201440.30.31.32), Muscular Dystrophy UK (17GRO- PS48-0093-1 and RA4/3023/1 with Duchenne Children's Trust and Duchenne Research Fund), IMI joint undertaking no. 115582 EBiSC, the UK MRC (MR/R014108/1, MR/L002752/1, and MR/J006785/1) and BBSRC (BB/J014567/1, BB/M009513/1, and BB/N503915/1), Duchenne Parent Project Onlus, and the AFM-Telethon (21687). S.T. was funded by Institut Pasteur and the Agence Nationale de la Recherche (Laboratoire d'Excellence Revive, Investissement d'Avenir; ANR-10-LABX-73 and ANR-16-CE12-0024). G.C. was supported by MRC (MR/P016006/1), GOSH-Sparks Charity (V4618) and Duchenne Parent Project Onlus. F.S.T. was funded by a National Institute for Health Research (NIHR) Academic Clinical Fellowship in Pediatrics (ACF-2015-18-001) and is grateful to F. Muntoni for his mentorship. P.D.C. and M.F.M.G. are also funded by the NIHR. The views expressed are those of the authors and not necessarily those of the National Health Service, the NIHR, or the Department of Health. The authors dedicate this work to the late Professor Paolo Bianco.

DECLARATION OF INTERESTS

F.S.T. was principal investigator on a research grant from Takeda New Frontier Science Program, received speaking and consulting fees from Takeda and Sanofi-Genzyme (via UCL Consultants), and has a collaboration with GSK (ICP BB/N503915/1).

Received: December 21, 2017
Revised: January 10, 2019
Accepted: January 11, 2019
Published: February 7, 2019

REFERENCES

Armulik, A., Abramsson, A., and Betsholtz, C. (2005). Endothelial/pericyte interactions. Circ. Res. *97*, 512–523.

Baghdadi, M.B., Castel, D., Machado, L., Fukada, S.I., Birk, D.E., Relaix, F., Tajbakhsh, S., and Mourikis, P. (2018). Reciprocal signalling by Notch-Collagen V-CALCR retains muscle stem cells in their niche. Nature *557*, 714–718.

Benedetti, S., Hoshiya, H., and Tedesco, F.S. (2013). Repair or replace? Exploiting novel gene and cell therapy strategies for muscular dystrophies. FEBS J. *280*, 4263–4280.

Betsholtz, C. (2004). Insight into the physiological functions of PDGF through genetic studies in mice. Cytokine Growth Factor Rev. *15*, 215–228.

Birbrair, A., and Delbono, O. (2015). Pericytes are essential for skeletal muscle formation. Stem Cell Rev. *11*, 547–548.

Bjornson, C.R., Cheung, T.H., Liu, L., Tripathi, P.V., Steeper, K.M., and Rando, T.A. (2012). Notch signaling is necessary to maintain quiescence in adult muscle stem cells. Stem Cells *30*, 232–242.

Boldrin, L., Muntoni, F., and Morgan, J.E. (2010). Are human and mouse satellite cells really the same? J. Histochem. Cytochem. *58*, 941–955.

Briggs, D., and Morgan, J.E. (2013). Recent progress in satellite cell/myoblast engraftment – relevance for therapy. FEBS J. *280*, 4281–4293.

Brohl, D., Vasyutina, E., Czajkowski, M.T., Griger, J., Rassek, C., Rahn, H.P., Purfurst, B., Wende, H., and Birchmeier, C. (2012). Colonization of the satellite cell niche by skeletal muscle progenitor cells depends on Notch signals. Dev. Cell *23*, 469–481.

Cappellari, O., Benedetti, S., Innocenzi, A., Tedesco, F.S., Moreno-Fortuny, A., Ugarte, G., Lampugnani, M.G., Messina, G., and Cossu, G. (2013). Dll4 and PDGF-BB convert committed skeletal myoblasts to pericytes without erasing their myogenic memory. Dev. Cell *24*, 586–599.

Cappellari, O., and Cossu, G. (2013). Pericytes in development and pathology of skeletal muscle. Circ. Res. *113*, 341–347.

Conboy, I.M., and Rando, T.A. (2002). The regulation of Notch signaling controls satellite cell activation and cell fate determination in postnatal myogenesis. Dev. Cell *3*, 397–409.

Cossu, G., Previtali, S.C., Napolitano, S., Cicalese, M.P., Tedesco, F.S., Nicastro, F., Noviello, M., Roostalu, U., Natali Sora, M.G., Scarlato, M., et al. (2015). Intra- arterial transplantation of HLA-matched donor mesoangioblasts in Duchenne muscular dystrophy. EMBO Mol. Med. *7*, 1513–1528.

Dellavalle, A., Sampaolesi, M., Tonlorenzi, R., Tagliafico, E., Sacchetti, B., Perani, L., Innocenzi, A., Galvez, B.G., Messina, G., Morosetti, R., et al. (2007). Pericytes of human skeletal muscle are myogenic precursors distinct from satellite cells. Nat. Cell Biol. *9*, 255–267.

Dellavalle, A., Maroli, G., Covarello, D., Azzoni, E., Innocenzi, A., Perani, L., Antonini, S., Sambasivan, R., Brunelli, S., Tajbakhsh, S., et al. (2011). Pericytes resident in postnatal skeletal muscle differentiate into muscle fibres and generate satellite cells. Nat. Commun. *2*, 499.

Esner, M., Meilhac, S.M., Relaix, F., Nicolas, J.F., Cossu, G., and Buckingham, M.E. (2006). Smooth muscle of the dorsal aorta shares a common clonal origin with skeletal muscle of the myotome. Development *133*, 737–749.

Gerli, M.F., Maffioletti, S.M., Millet, Q., and Tedesco, F.S. (2014). Transplantation of induced pluripotent stem cell-derived mesoangioblast-like myogenic progenitors in mouse models of muscle regeneration. J. Vis. Exp., e50532.

Giannotta, M., Benedetti, S., Tedesco, F.S., Corada, M., Trani, M., D'Antuono, R., Millet, Q., Orsenigo, F., Galvez, B.G., Cossu, G., et al. (2014). Targeting endothelial junctional adhesion molecule-A/EPAC/Rap-1 axis as a novel strategy to increase stem cell engraftment in dystrophic muscles. EMBO Mol. Med. *6*, 239–258.

Goupille, O., Pallafacchina, G., Relaix, F., Conway, S.J., Cumano, A., Robert, B., Montarras, D., and Buckingham, M. (2011). Characterization of Pax3-expressing cells from adult blood vessels. J. Cell Sci. *124*, 3980–3988.

Guimaraes-Camboa, N., Cattaneo, P., Sun, Y., Moore-Morris, T., Gu, Y., Dalton, N.D., Rockenstein, E., Masliah, E., Peterson, K.L., Stallcup, W.B., et al. (2017). Pericytes of multiple organs do not behave as mesenchymal stem cells in vivo. Cell Stem Cell *20*, 345–359.e5.

Jin, S., Hansson, E.M., Tikka, S., Lanner, F., Sahlgren, C., Farnebo, F., Baumann, M., Kalimo, H., and Lendahl, U. (2008). Notch signaling regulates platelet-derived growth factor receptor-beta expression in vascular smooth muscle cells. Circ. Res. *102*, 1483–1491.

Kopan, R., Nye, J.S., and Weintraub, H. (1994). The intracellular domain of mouse Notch: a constitutively activated repressor of myogenesis directed at the basic helix-loop-helix region of MyoD. Development *120*, 2385–2396.

Kostallari, E., Baba-Amer, Y., Alonso-Martin, S., Ngoh, P., Relaix, F., Lafuste, P., and Gherardi, R.K. (2015). Pericytes in the myovascular niche promote post-natal myofiber growth and satellite cell quiescence. Development *142*, 1242–1253.

Lagha, M., Brunelli, S., Messina, G., Cumano, A., Kume, T., Relaix, F., and Buckingham, M.E. (2009). Pax3:Foxc2 reciprocal repression in the somite modulates muscular versus vascular cell fate choice in multipotent progenitors. Dev. Cell *17*, 892–899.

Lindahl, P., Johansson, B.R., Leveen, P., and Betsholtz, C. (1997). Pericyte loss and microaneurysm formation in PDGF-B-deficient mice. Science *277*, 242–245.

Loperfido, M., Steele-Stallard, H.B., Tedesco, F.S., and VandenDriessche, T. (2015). Pluripotent stem cells for gene therapy of degenerative muscle diseases. Curr. Gene Ther. *15*, 364–380.

Low, S., Barnes, J.L., Zammit, P.S., and Beauchamp, J.R. (2018). Delta-like 4 activates notch 3 to regulate self-renewal in skeletal muscle stem cells. Stem Cells *36*, 458–466.

Lu, W., and Li, X. (2017). PDGFs and their receptors in vascular stem/progenitor cells: functions and therapeutic potential in retinal vasculopathy. Mol. Aspects Med. *62*, 22–32.

Madisen, L., Zwingman, T.A., Sunkin, S.M., Oh, S.W., Zariwala, H.A., Gu, H., Ng, L.L., Palmiter, R.D., Hawrylycz, M.J., Jones, A.R., et al. (2010). A robust and high-throughput Cre reporting and characterization system for the whole mouse brain. Nat. Neurosci. *13*, 133–140.

Mourikis, P., Sambasivan, R., Castel, D., Rocheteau, P., Bizzarro, V., and Tajbakhsh, S. (2012). A critical requirement for notch signaling in maintenance of the quiescent skeletal muscle stem cell state. Stem Cells *30*, 243–252.

Mourikis, P., and Tajbakhsh, S. (2014). Distinct contextual roles for Notch signalling in skeletal muscle stem cells. BMC Dev. Biol. *14*, 2.

Murray, I.R., Baily, J.E., Chen, W.C., Dar, A., Gonzalez, Z.N., Jensen, A.R., Petrigliano, F.A., Deb, A., and Henderson, N.C. (2017). Skeletal and cardiac muscle pericytes: functions and therapeutic potential. Pharmacol. Ther. *171*, 65–74.

Okabe, M., Ikawa, M., Kominami, K., Nakanishi, T., and Nishimune, Y. (1997). “Green mice” as a source of ubiquitous green cells. FEBS Lett. *407*, 313–319.

Pallafacchina, G., Francois, S., Regnault, B., Czarny, B., Dive, V., Cumano, A., Montarras, D., and Buckingham, M. (2010). An adult tissue-specific stem cell in its niche: a gene profiling analysis of in vivo quiescent and activated muscle satellite cells. Stem Cell Res. *4*, 77–91.

Parker, M.H., Loretz, C., Tyler, A.E., Duddy, W.J., Hall, J.K., Olwin, B.B., Bernstein, I.D., Storb, R., and Tapscott, S.J. (2012). Activation of Notch signaling during ex vivo expansion maintains donor muscle cell engraftment. Stem Cells *30*, 2212–2220.

Partridge, T. (2002). Myoblast transplantation. Neuromuscul. Disord. *12* (*Suppl 1*), S3–S6.

Perie, S., Trollet, C., Mouly, V., Vanneaux, V., Mamchaoui, K., Bouazza, B., Marolleau, J.P., Laforet, P., Chapon, F., Eymard, B., et al. (2014). Autologous myoblast transplantation for oculopharyngeal muscular dystrophy: a phase I/IIa clinical study. Mol. Ther. *22*, 219–225.

Pinol-Jurado, P., Gallardo, E., de Luna, N., Suarez-Calvet, X., Sanchez-Riera, C., Fernandez-Simon, E., Gomis, C., Illa, I., and Diaz-Manera, J. (2017). Platelet-derived growth factor BB influences muscle regeneration in Duchenne muscle dystrophy. Am. J. Pathol. *187*, 1814–1827.

Quattrocelli, M., Costamagna, D., Giacomazzi, G., Camps, J., and Sampaolesi, M. (2014). Notch signaling regulates myogenic regenerative capacity of murine and human mesoangioblasts. Cell Death Dis. *5*, e1448.

Relaix, F., and Zammit, P.S. (2012). Satellite cells are essential for skeletal muscle regeneration: the cell on the edge returns centre stage. Development *139*, 2845–2856.

Sacchetti, B., Funari, A., Remoli, C., Giannicola, G., Kogler, G., Liedtke, S., Cossu, G., Serafini, M., Sampaolesi, M., Tagliafico, E., et al. (2016). No identical “mesenchymal stem cells” at different times and sites: human committed progenitors of distinct origin and differentiation potential are incorporated as adventitial cells in microvessels. Stem Cell Reports *6*, 897–913.

Sainson, R.C., and Harris, A.L. (2008). Regulation of angiogenesis by homotypic and heterotypic notch signalling in endothelial cells and pericytes: from basic research to potential therapies. Angiogenesis *11*, 41–51.

Sakai, H., Fukuda, S., Nakamura, M., Uezumi, A., Noguchi, Y.T., Sato, T., Morita, M., Yamada, H., Tsuchida, K., Tajbakhsh, S., et al. (2017). Notch ligands regulate the muscle stem-like state ex vivo but are not sufficient for retaining regenerative capacity. PLoS One *12*, e0177516.

Sambasivan, R., Gayraud-Morel, B., Dumas, G., Cimper, C., Paisant, S., Kelly, R.G., and Tajbakhsh, S. (2009). Distinct regulatory cascades govern extraocular and pharyngeal arch muscle progenitor cell fates. Dev. Cell *16*, 810–821.

Sambasivan, R., and Tajbakhsh, S. (2015). Adult skeletal muscle stem cells. Results Probl. Cell Differ. *56*, 191–213.

Schuster-Gossler, K., Cordes, R., and Gossler, A. (2007). Premature myogenic differentiation and depletion of progenitor cells cause severe muscle hypotrophy in Delta1 mutants. Proc. Natl. Acad. Sci. U S A *104*, 537–542.

Skuk, D., and Tremblay, J.P. (2014). Clarifying misconceptions about myoblast transplantation in myology. Mol. Ther. *22*, 897–898.

Srinivas, S., Watanabe, T., Lin, C.S., William, C.M., Tanabe, Y., Jessell, T.M., and Costantini, F. (2001). Cre reporter strains produced by targeted insertion of EYFP and ECFP into the ROSA26 locus. BMC Dev. Biol. *1*, 4.

Sugg, K.B., Korn, M.A., Sarver, D.C., Markworth, J.F., and Mendias, C.L. (2017). Inhibition of platelet-derived growth factor signaling prevents muscle fiber growth during skeletal muscle hypertrophy. FEBS Lett. *591*, 801–809.

Tedesco, F.S., Dellavalle, A., Diaz-Manera, J., Messina, G., and Cossu, G. (2010). Repairing skeletal muscle: regenerative potential of skeletal muscle stem cells. J. Clin. Invest. *120*, 11–19.

Tedesco, F.S., Gerli, M.F., Perani, L., Benedetti, S., Ungaro, F., Cassano, M., Antonini, S., Tagliafico, E., Artusi, V., Longa, E., et al. (2012). Transplantation of genetically corrected human iPSC-derived progenitors in mice with limb-girdle muscular dystrophy. Sci. Transl. Med. *4*, 140ra89.

Tedesco, F.S., Hoshiya, H., D’Antona, G., Gerli, M.F., Messina, G., Antonini, S., Tonlorenzi, R., Benedetti, S., Berghella, L., Torrente, Y., et al. (2011). Stem cell-mediated transfer of a human artificial chromosome ameliorates muscular dystrophy. Sci. Transl. Med. *3*, 96ra78.

Tedesco, F.S., Moyle, L.A., and Perdiguero, E. (2017). Muscle interstitial cells: a brief field guide to non-satellite cell populations in skeletal muscle. Methods Mol. Biol. *1556*, 129–147.

Vasyutina, E., Lenhard, D.C., Wende, H., Erdmann, B., Epstein, J.A., and Birchmeier, C. (2007). RBP-J (Rbpsuh) is essential to maintain muscle progenitor cells and to generate satellite cells. Proc. Natl. Acad. Sci. U S A *104*, 4443–4448.

Verma, M., Asakura, Y., Murakonda, B.S.R., Pengo, T., Latroche, C., Chazaud, B., McLoon, L.K., and Asakura, A. (2018). Muscle satellite cell cross-talk with a vascular niche maintains quiescence via VEGF and notch signaling. Cell Stem Cell. *23*, 530–543.

Xu, J., Du, Y., and Deng, H. (2015). Direct lineage reprogramming: strategies, mechanisms, and applications. Cell Stem Cell *16*, 119–134.

Yablonka-Reuveni, Z., Balestreri, T.M., and Bowen-Pope, D.F. (1990). Regulation of proliferation and differentiation of myoblasts derived from adult mouse skeletal muscle by specific isoforms of PDGF. J. Cell Biol. *111*, 1623–1629.

Zammit, P.S., Golding, J.P., Nagata, Y., Hudon, V., Partridge, T.A., and Beauchamp, J.R. (2004). Muscle satellite cells adopt divergent fates: A mechanism for self-renewal? J. Cell Biol. *166*, 347–357.

Stem Cell Reports, Volume *12*

Supplemental Information

Combined Notch and PDGF Signaling Enhances Migration and Expression of Stem Cell Markers while Inducing Perivascular Cell Features in Muscle Satellite Cells

Mattia Francesco Maria Gerli, Louise Anne Moyle, Sara Benedetti, Giulia Ferrari, Ekin Ucuncu, Martina Ragazzi, Chrystalla Constantinou, Irene Louca, Hiroshi Sakai, Pierpaolo Ala, Paolo De Coppi, Shahragim Tajbakhsh, Giulio Cossu, and Francesco Saverio Tedesco

SUPPLEMENTAL INFORMATION

Combined Notch and PDGF Signaling Enhances Migration and Expression of Stem Cell Markers while Inducing Perivascular Cell Features in Muscle Satellite Cells

Mattia Francesco Maria Gerli, Louise Anne Moyle, Sara Benedetti, Giulia Ferrari, Ekin Ucuncu, Martina Ragazzi, Chrystalla Constantinou, Irene Louca, Hiroshi Sakai, Pierpaolo Ala, Paolo De Coppi, Shahragim Tajbakhsh, Giulio Cossu and Francesco Saverio Tedesco

INVENTORY:

- **SUPPLEMENTAL FIGURES**
    - **Figure S1.** Additional Analyses of DLL4 & PDGF-BB Treated Adult Murine SCs, Related to Figure 2.
    - **Figure S2.** Migration of Treated SCs within Subcutaneous Matrigel Plugs, Related to Figure 3.
- **SUPPLEMENTAL EXPERIMENTAL PROCEDURES**
    - Cell isolation, culture and differentiation
    - Morphological and proliferation analyses
    - Network formation and migration assays
    - Cell transplantation
    - Sequences of primers used for RT-qPCR
- **SUPPLEMENTAL REFERENCES**

SUPPLEMENTAL FIGURES

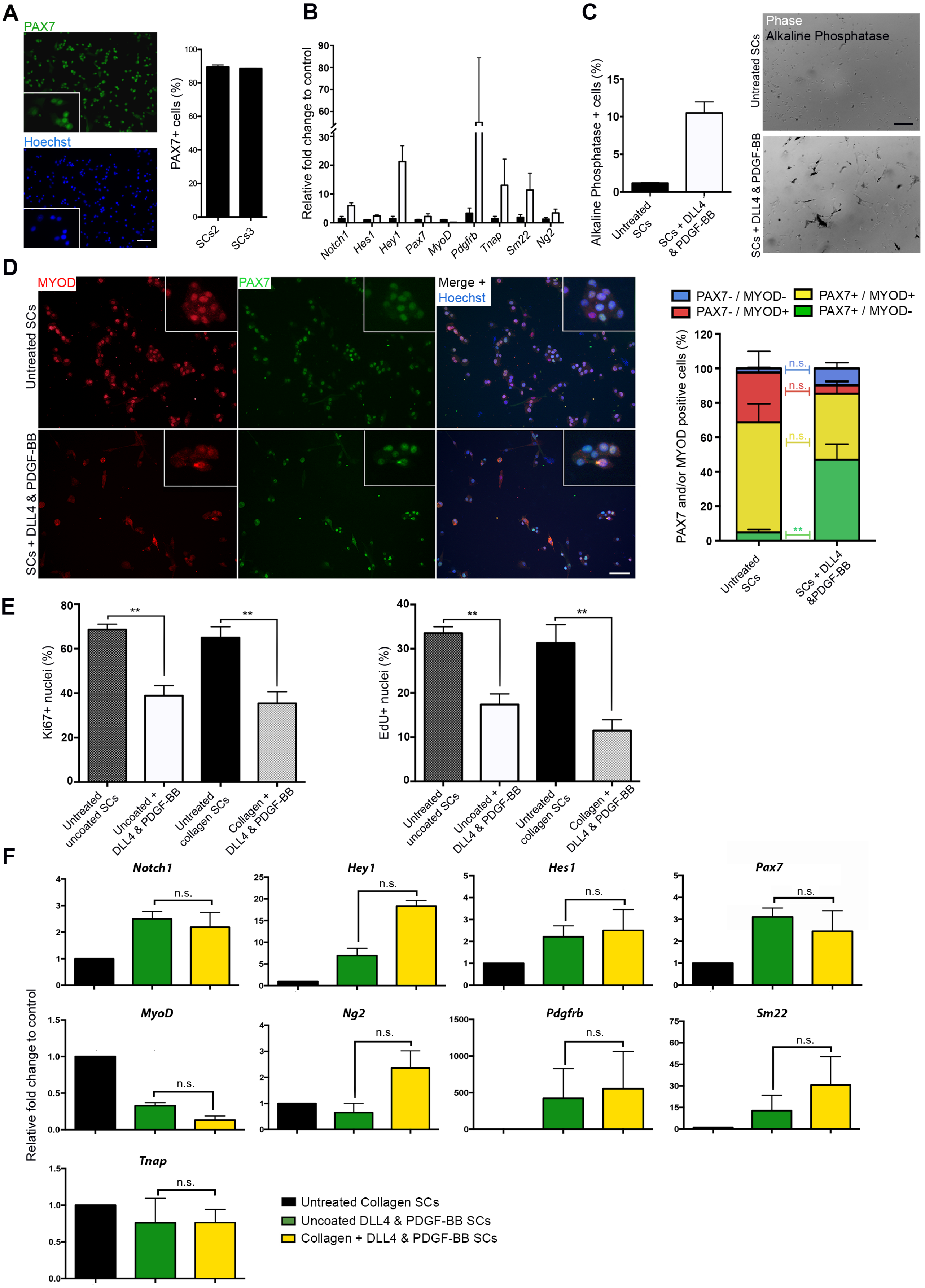

Figure S1. Additional analyses of DLL4 & PDGF-BB-treated adult murine SCs; related to Figure 2. (A). Left: representative immunofluorescence panel showing PAX7 (green) staining on murine SCs isolated with the pre-plating technique and cultured for 2 passages after isolation before staining; Hoechst (blue): nuclei. Right: graph quantifying the percentage of PAX7+ cells in two SC preparations (N = 3, a minimum of 500 nuclei were counted per preparations). **(B)** RT-qPCR expression analysis of SC genes (*Pax7, MyoD*), smooth muscle genes (*Tnap, Ng2,* and *Sm22*) and Notch and PDGF signaling effectors (*Notch1, Hes1*, *Hey1 and Pdgfrb*) in control (black bars) and DLL4 & PDGF-BB-treated (white bars) SCs from CD1 mice. Graphs show fold change to control, statistical significance based on ΔCt (n = 3). **(C)** Alkaline phosphatase activity in untreated and treated SCs (n = 3). **(D)** Immunofluorescence images of SCs expanded for 2 weeks prior to DLL4 & PDGF-BB treatment and co-immunostained with antibodies against PAX7 (green) and MYOD (red). The relative percentages of PAX7+/MYOD-, PAX7+/MYOD +, PAX7-/MYOD+ and PAX7-/MYOD- cells were quantified (N = 4). **(E)** Treatment of PAX7-GFP+ SCs on uncoated or collagen-coated dishes (N = 3). Left graph shows the percentage of Ki67+ cells decreasing irrespective of coating in response to treatment: from 64.94% in untreated collagen to 35.40% in collagen + DLL4 & PDGF-BB (p = 0.023), and from 70.79 % in untreated collagen to 42.50% in uncoated + DLL4 & PDGF-BB ($p = 0.044$). Right graph shows the percentage of EdU+ cells decreasing irrespective of coating in response to treatment: from 31.30% in untreated collagen to 11.49% in collagen + DLL4 & PDGF-BB ($p = 0.006$), and from 34.20% in untreated collagen to 18.60% in uncoated + DLL4 & PDGF-BB ($p = 0.002$). **(F)** RT-qPCR expression analysis of the same genes shown in (B) in response to DLL4 & PDGF-BB treatment on collagen-coated or uncoated dishes (no difference observed). Graphs presented as mean ± SEM and statistical significance based on two-way ANOVA with Bonferroni's (C) or Tukey's (D, F) multiple comparison, or a paired student's *t*-test (E). $^{*}p < 0.05$, $^{**}p < 0.01$. Scale bars 100 μm (B) and 50 μm (A, C).

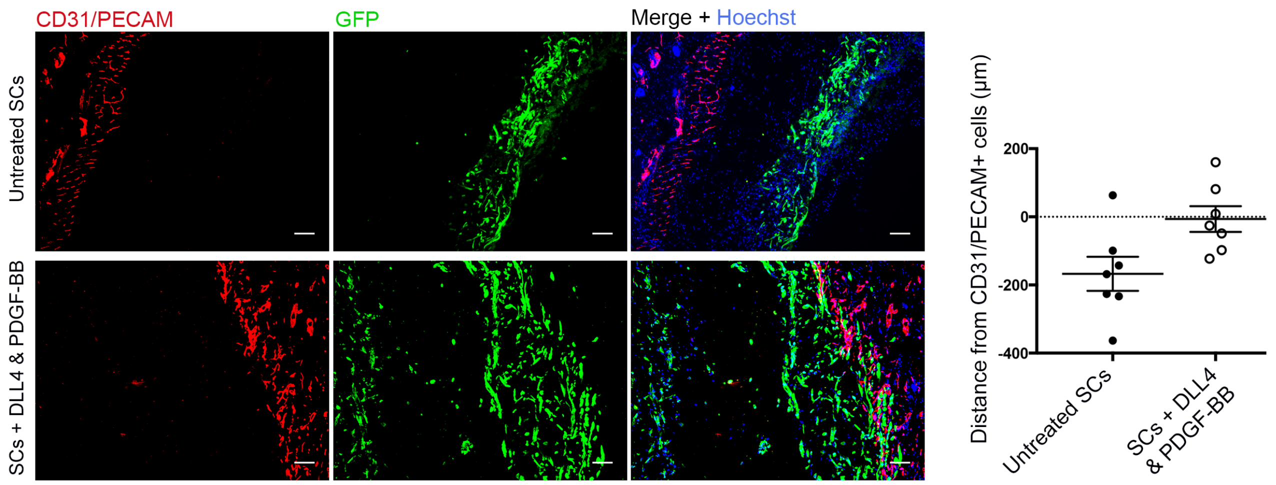

Figure S2. Migration of treated SCs within subcutaneous Matrigel plugs; related to Figure 3. Representative images of *in vivo* Matrigel plugs from mice injected with untreated (top panel) or DLL4 & PDGF-BB-treated SCs (lower panel) immunostained for PECAM/CD31 (red), GFP (green) and Hoechst nuclear stain (blue). The distance between GFP+ SCs and CD31/PECAM+ cells of the host vasculature was calculated (negative values = distance away from nearest blood vessel, 0 = in contact, positive values = distance inside the host vasculature bed) (N = 2). Scale bars 100 μm.

SUPPLEMENTAL EXPERIMENTAL PROCEDURES

Cell isolation, culture and differentiation

Primary SCs were isolated from dissected adult muscles by mechanical fragmentation and digested twice in 0.1% Collagenase D (Roche) + 0.24U/ml Dispase (Gibco) + 0.1 mg/ml DNase (Roche), diluted in DMEM (Sigma-Aldrich) for 20 minutes at 37°C in agitation. The digestion product was filtered through a 40 μm cell strainer (Thermo Fisher) and enzyme activity inhibited with 20% fetal bovine serum (FBS; Life Technologies). The mononuclear cell suspension was washed twice in phosphate buffered saline (PBS) and pre-plated on cell culture dishes at 37°C 5% CO_2 for 30 minutes to remove fibroblast contamination and enrich the floating fraction of less adherent SCs. The supernatant was collected and transferred to dishes coated with collagen (C8919 from calf skin Sigma-Aldrich) at a density

of 1.2 x 10^3 cells/cm^2. Mononuclear cell suspensions from 6 to 8-week old female *Tg:Pax7-nGFP* mice were FACS-purified in PBS + 2% FBS + 2mM EDTA (Invitrogen) on a MoFlo XDP cell sorter (Beckman Coulter).

Primary murine myogenic pericytes were isolated based on TN-AP expression as previously reported (Dellavalle et al., 2011). Briefly, *TN-AP-CreERT2* mice were crossed with Td: Tomato or $R26R^{EYFP}$ lines and resulting litters subcutaneously injected daily with 0.3 mg tamoxifen (Sigma-Aldrich) from P6-8 to induce Cre-recombination. Muscles were harvested at P19-21, digested as described above and pericytes FACS-purified on a FACS Aria III (BD Biosciences) or MoFlo XDP (Beckman Coulter).

Primary murine SCs and muscle pericytes were cultured at low density on collagen-coated dishes at 37°C, 3% O_2 and 5% CO_2 in primary murine growth medium: DMEM-F/12 (Gibco, USA) supplemented with 20% FBS , 10% horse serum (HS)(Euroclone), 1% non-essential amino acids (NEAA; Sigma-Aldrich), 1 mM sodium-pyruvate (Sigma-Aldrich), 1% insulin-transferrin-selenium (Sigma-Aldrich), 2 mM glutamine (Sigma-Aldrich), 50 μM β-mercaptoethanol (Gibco), 1% penicillin-streptomycin (P/S; Sigma-Aldrich), 50 µg/ml gentamycin (Gibco) and 5 ng/ml bFGF (Gibco).

Primary human SC-derived myoblasts (HuSCs) were obtained from the MRC Neuromuscular Centre Biobank and tissue sampling was conducted under the approval of the NHS Health Research Authority Research Ethics Committee (NHS HRA REC) reference no. 06/Q0406/33. Human cell work conducted under NHS HRA REC no. 13/LO/1826, IRAS project ID no. 141100. To increase purity, HuSCs were isolated by FACS for CD56+ (Biolegend; CD56-FITC 304604). Cells were cultured at low density in MegaCell DMEM medium (Sigma-Aldrich) supplemented with 5% FBS, 2 mM glutamine, 1% NEAA, 1% P/S, 50 µM β-mercaptoethanol and 5ng/ml bFGF. Primary human pericyte-derived mesoangioblasts (MABs) were the 27XY preparation from (Bonfanti et al., 2015), which were cultured in the same MegaCell-based medium as HuSCs.

C2C12 murine myoblasts (used to produce conditioned-medium) (Yaffe and Saxel, 1977) and H5V murine endothelial cells (Garlanda et al., 1994) were cultured at 37°C, 3% O_2, 5% CO_2 in DMEM supplemented with 10% FBS, 2 mM glutamine and 1% P/S. Primary human umbilical vein endothelial cells (HUVECs; Lonza) were maintained at 37°C, 5% CO_2 in EGM1 (Lonza) on 1% gelatin-coated flasks (Sigma-Aldrich). HUVECs were kept below 70% confluence and used until passage 6.

For myogenic differentiation assays, cells were seeded at a high density (1.2 x 10^4 cells/cm^2) on collagen-coated dishes and grown until confluence, then switched to differentiation medium (DMEM supplemented with 2% HS + 1% P/S), replaced every other day for 4 days before fixation. To block Notch signaling, samples were incubated with 660 ng/ml L-685,458 (γ-secretase inhibitor; Sigma-Aldrich) 24 hours prior to the medium-switch and added to each subsequent medium change.

Morphological and proliferation analyses

Phase contrast images of proliferating cells were analyzed to measure the cell circularity ratio (1 = circle; 0 = line). The cell contours were drawn and quantified using Image J software. A minimum of 500 cells were analyzed from 3 random fields per condition, and each experiment was performed with at least 3 independent repeats.

To measure proliferation rate, cells were pulsed for 2 hours with 10 μM 5- Ethynyl-2'-deoxyuridine (EdU) before fixation and processing following manufacturer's instructions (Click-iT® EdU Alexa Fluor® Imaging Kit, Life Technologies). To generate growth curves cells were cultured for three weeks, trypsinized every other day and counted using Glasstic slides (Kova; 87144).

Network formation and migration assays

For endothelial network formation assays, 24 multi-well dishes were coated for 30 minutes

at 37°C with growth factor-reduced Matrigel (Corning) diluted 1:1 in EGM1 medium (Lonza). 5 x 10^4 HUVECs/cm^2 were then seeded on top of the Matrigel-coated dishes, followed after 30 minutes by 10^4 GFP+ SCs or pericytes from *Tg:TN-AP-CreERT2:R26R-EYFP* mice (ratio of 1:10 to HUVECs), together with 10 ng/ml of recombinant human vascular endothelial growth factor (VEGF, R&D Systems). After 24 hours the number of network branches / high-power field (HPF; 10X lens = 1.5 mm^2) was quantified with an inverted fluorescence microscope (Leica). For quantification, 2-5 random pictures per well were taken and the data displayed as numbers of network branches/mm^2. Four independent experiments were performed by 4 independent operators, with each sample repeated in duplicate in at least 3 experiments. Association of fluorescent cells with the HUVEC network was also monitored over time up to 120 hours post-seeding.

For murine cell migration experiments, H5V endothelial cells were grown to confluence on 8 μm porous cell culture membranes (BD Falcon 353093) coated with 1% gelatin for 1 hour at 37°C. To assess trans-endothelial migration, 1 x 10^4 GFP+ SCs, *TN-AP:YFP*+ primary pericytes or C57 *GFP*+/*nLacZ*+ mesoangioblasts were seeded in the upper transwell chamber in serum-free medium. The lower chamber was filled with 50% growth medium + 50% C2C12 myotube-conditioned medium. After 6 hours, membranes were fixed for 5 minutes in 4% PFA and cells from the upper side of the membrane removed using a cell scraper. The average number of migrated cells was quantified from at least five 10X fields (1.5 mm^2). Transwell assays with human cells were performed as previously described (Giannotta et al., 2014). 2 x 10^5 HUVECs were seeded for 72 hours. Human SCs and mesoangioblasts were dissociated with TrypLE and labelled with 0.7 μM 6-carboxyfluorescin dictate (both Thermo Fisher Scientific) for 30 minutes at 37°C and 5 x 10^4 cells seeded per membrane for 8 hours before processing and quantifying as described above.

Cell transplantation

Three to five-month-old *sgca-null/scid/beige* mice were used for transplantation experiments, which were performed as previously described (Gerli et al., 2014). Briefly, SCs from *Tg:CAG-EGFP* mice (Okabe et al., 1997) were transduced with a lentivirus encoding nuclear β-galactosidase (*nLacZ*) (Tedesco et al., 2011) and then expanded. For intramuscular injections, cells were trypsinized and resuspended in Ca^{2+}/Mg^{2+}-free PBS to a concentration of 1 x 10^6 cells/30 μl and injected with a 30G needle into tibialis anterior muscles (N = 5/condition). Two muscles were sham injected (PBS). For intra-arterial delivery, cells were filtered through a 40 μm strainer, re-suspended at a concentration of 1 x 10^6 cells/50 μl, and injected into femoral arteries using a 30G needle (N = 7/condition). Contralateral limbs were injected with control SCs and 2 limbs were sham injected (PBS). Muscles were harvested after 23 days and assessed for the presence of donor-derived cells, either as whole mounts or flash-frozen in isopentane-cooled in liquid nitrogen, then serially cryosectioned using a Leica CM 1850 UV cryostat. Analysis of transplantation was performed blinded, and intra-arterial transplantations were normalized to the volume of cells injected.

Tumorigenic assays were performed as previously reported (Tedesco et al., 2012). Briefly, 2 x 10^6 cells were harvested, re-suspended in 100μl of PBS and injected subcutaneously in immunodeficient scid/beige mice (N = 5). Animals were observed bi-weekly and euthanized after 2 months to confirm the absence of masses.

For subcutaneous plugs, cells were mixed 1:1 with HUVECs in Matrigel supplemented with 10 ng/ml VEGF (Sigma-Aldrich) and slowly injected subcutaneously into two 3-month-old nude mice using refrigerated syringes. Animals were anesthetized for 15 minutes to facilitate Matrigel polymerization. After 2 weeks, plugs were harvested, frozen in liquid nitrogen-cooled isopentane and cryosectioned.

Sequences of primers used for RT-qPCR

Name	Forward sequence 5'-3'	Reverse Sequence 5'-3'
Gapdh	TTCACCACCATGGAGAAGGC	GGCATGGACTGTGGTCATGA
Notch1	TGGACGCCGCTGTGAGTCA	TGGGCCCGAGATGCATGTA
Hes1	ACACCGGACAAACCAAAGAC	AATGCCGGGAGCTATCTTTC
Hey1	CACCTGAAAATGCTGCACAC	ATGCTCAGATAACGGGCAAC
Pax7	ATGTTCAGCTGGGAAATCCGGG	TCCCGAACTTGATTCTGAGCACTCG
MyoD	GCCCGCGCTCCAACTGCTCTGAT	GCCCGCGCTCCAACTGCTCTGAT
Pdgfrb	GCTCACGGTCTGAGCCATTC	GCTCGGACATTAAGGCTTGCT
Sm22	CCAACAAGGGTCCATCCTACG	ATCTGGGCGGCCTACATCA
Ng2	ACAAGCGTGGCAACTTTATC	ATAGACCTCTTCTTCATATT
Tnap	GTGGATACACCCCCCCGGGGC	GGTCAAGGTGGCCCCAATGCA
RPLPO	TCTACAACCCTGAAGTGCTTGAT	CAATCTGCAGACAGACACTGG
PAX7	CAAACACAGCATCGACGG	CTTCAGTGGGAGGTCAGGTT
MYOD	CACGTCGAGCAATCCAAACC	TGTAGTCCATCATGCCGTCG
MYOD	AATAAGAGTTGCTTTGCCAG	GTACAAATTCCCTGTAGCAC
MYOGENIN	CCAGGGGTGCCCAGCGAATG	AGCCGTGAGCAGATGATCCCC
PDGFRB	AGCTGTTACCCACTCTGGGA	TGGTGTCCTTGCTGCTGATG
TNAP	TGTGGGGTGAAGGCCAATG	GTGGTGGTCACAATGCCCA
CD146	GGAAGCAGGAGATCACGCTAC	GATTCGGGGCTAATGCCTCA
HEY1	AGGTTACTTTGACGCGCACG	ACCAGTCGAACTCGAAGCG
HES1	AGAAAGATAGCTCGCGGCA	TACTTCCCCAGCACACTTGG

SUPPLEMENTAL REFERENCES

Bonfanti, C., Rossi, G., Tedesco, F.S., Giannotta, M., Benedetti, S., Tonlorenzi, R., Antonini, S., Marazzi, G., Dejana, E., Sassoon, D., *et al.* (2015). PW1/Peg3 expression regulates key properties that determine mesoangioblast stem cell competence. Nat Commun *6*, 6364.

Garlanda, C., Parravicini, C., Sironi, M., De Rossi, M., Wainstok de Calmanovici, R., Carozzi, F., Bussolino, F., Colotta, F., Mantovani, A., and Vecchi, A. (1994). Progressive growth in immunodeficient mice and host cell recruitment by mouse endothelial cells transformed by polyoma middle-sized T antigen: implications for the pathogenesis of opportunistic vascular tumors. Proc Natl Acad Sci U S A *91*, 7291-7295.

Yaffe, D., and Saxel, O. (1977). Serial passaging and differentiation of myogeniccells isolated from dystrophic mouse muscle. Nature *270*, 725-727.
